# Insulin-Like Peptides Regulate Feeding Preference and Metabolism in *Drosophila*

**DOI:** 10.3389/fphys.2018.01083

**Published:** 2018-08-24

**Authors:** Uliana V. Semaniuk, Dmytro V. Gospodaryov, Khrystyna M. Feden'ko, Ihor S. Yurkevych, Alexander M. Vaiserman, Kenneth B. Storey, Stephen J. Simpson, Oleh Lushchak

**Affiliations:** ^1^Department of Biochemistry and Biotechnology, Vasyl Stefanyk Precarpathian National University, Ivano-Frankivsk, Ukraine; ^2^D.F. Chebotarev Institute of Gerontology, NAMS, Kiev, Ukraine; ^3^Department of Biology, Institute of Biochemistry, Carleton University, Ottawa, ON, Canada; ^4^Charles Perkins Centre, University of Sydney, Sydney, NSW, Australia

**Keywords:** geometric framework, dietary response surface, macronutrient balance, nutrient intake trajectories, capillary feeding, *Drosophila* insulin-like peptides

## Abstract

Fruit flies have eight identified *Drosophila* insulin-like peptides (DILPs) that are involved in the regulation of carbohydrate concentrations in hemolymph as well as in accumulation of storage metabolites. In the present study, we investigated diet-dependent roles of DILPs encoded by the genes *dilp1*–*5*, and *dilp7* in the regulation of insect appetite, food choice, accumulation of triglycerides, glycogen, glucose, and trehalose in fruit fly bodies and carbohydrates in hemolymph. We have found that the wild type and the mutant lines demonstrate compensatory feeding for carbohydrates. However, mutants on *dilp2,3, dilp3, dilp5*, and *dilp7* showed higher consumption of proteins on high yeast diets. To evaluate metabolic differences between studied lines on different diets we applied response surface methodology. High nutrient diets led to a moderate increase in concentration of glucose in hemolymph of the wild type flies. Mutations on *dilp* genes changed this pattern. We have revealed that the *dilp2* mutation led to a drop in glycogen levels independently on diet, lack of *dilp3* led to dramatic increase in circulating trehalose and glycogen levels, especially at low protein consumption. Lack of *dilp5* led to decreased levels of glycogen and triglycerides on all diets, whereas knockout on *dilp7* caused increase in glycogen levels and simultaneous decrease in triglyceride levels at low protein consumption. Fruit fly appetite was influenced by *dilp3* and *dilp7* genes. Our data contribute to the understanding of *Drosophila* as a model for further studies of metabolic diseases and may serve as a guide for uncovering the evolution of metabolic regulatory pathways.

## Introduction

The fruit fly, *Drosophila melanogaster*, serves as a model for investigating the molecular mechanisms underlying various pathological states such as type II diabetes, obesity, and metabolic syndrome. These conditions involve dysregulation of metabolism and share evolutionarily conserved genetic and environmental determinants (Leulier et al., 2017). Fruit flies and humans have common molecular regulators of key metabolic pathways, including hormones, transcriptional factors, and signaling molecules that govern cellular and organ metabolism. Insulin plays a special role among these regulators, controlling many aspects of carbohydrate and lipid metabolism, and disorders in insulin signaling and regulation cause diabetes. *Drosophila* insulin-like peptides (DILPs) regulate the balance between stored and circulating carbohydrates and also regulate metabolism by triggering signaling cascades that affect transcription of genes responsible for metabolic reconfigurations. Generally, DILPs promote accumulation of glycogen and fat stores (DiAngelo and Birnbaum, 2009), suppress gluconeogenesis but induce glycolysis in cells (Musselman et al., 2011), and control growth and suppress food intake homeostatically when a sufficient amount of food is ingested (Nässel et al., 2013). The disruption of DILP regulation leads to multiple defects of growth and development. Many recent investigations have revealed complex interactions between insulin signaling and other hormonal signals in *Drosophila*. Particularly, relationships have been shown between DILPs and developmental hormones of insects (Géminard et al., 2006), as well as small peptides which regulate appetite (Nässel et al., 2013). The release of DILPs is, in turn, controlled by neuropeptides with reverse feedback regulation (Nässel et al., 2013).

Unlike human insulin signaling, *D. melanogaster* has eight DILPs that are produced in different conditions or tissues, having diverse roles (Nässel et al., 2013). Knockdowns of genes coding DILPs in *D. melanogaster* result in various growth defects (Zhang et al., 2009) and removal of insulin-producing cells in fruit flies produces a phenocopy of type I diabetes in humans (Rulifson et al., 2002). However, this has not yet been studied under different dietary conditions, where diet might modulate the phenotype of DILPs deficiency. Macronutrient combinations, as well as the quality of macronutrients and interactions with micronutrients, can modulate disease outcomes, thus offering the opportunity for diet to serve as both prevention and cure (Simpson et al., 2015, 2017; Raubenheimer and Simpson, 2016). We have shown recently in flies that macronutrient balance defines physiological parameters such as fecundity and stress resistance (Lushchak et al., 2014) and may also alter the impact of plant medicinal preparations (Gospodaryov et al., 2013). The goal of the current study was to systematically vary dietary macronutrient balance in combination with specific DILP knockouts to assess the effects on appetite and nutrient preferences of flies as well as on stored and circulating carbohydrates. This has allowed us to begin to dissect the roles of individual DILP types and to explore the dietary conditions under which different DILPs are released and operate.

## Materials and methods

### Fruit fly lines

*Drosophila melanogaster* flies were cultured on standard medium (7.5% molasses, 5% yeast, 6% corn, 1% agar, and 0.18 nipagin as a mold inhibitor) at 25°C, 12:12 light:dark cycle and 60% relative humidity. Flies of the *w*^1118^ wild type line and mutants generated on the *w*^1118^ background for *dilp1, dilp2, dilp2,3, dilp3, dilp4, dilp5*, and *dilp7* were kindly provided by Dr. Sebastian Grönke (Max Planck Institute for Biology of Aging, Cologne, Germany; Table S1; Grönke et al., 2010). The lines were backcrossed to the wild type for more than 10 generations just before the start of experiments (Dr. Sebastian Grönke, personal communication).

### Feeding assays

For experiments, 2-day-old flies were transferred to fresh food and kept for an additional 48 h for mating. Females were collected under light CO_2_ anesthesia and placed into plastic tubes (1.5 ml) with three holes for ventilation, and two holes in the cap for the positioning of 5 μl capillary feeding tubes (Drummond Scientific Company) filled with nutrient solution, either sucrose (Carl Roth GmbH & Co., #9286) or yeast autolysate (MP Biomedicals, #02103304). Phosphoric (0.01%) and propionic (0.1%) acids were added to both solutions as inhibitors of bacteria and mold growth. Tubes with flies were placed into closed containers with moistened cotton tissue in the bottom to maintain high humidity and decrease evaporation of solutions from the capillaries. Tubes with capillaries but without the flies were used as the evaporation controls, and those values were subtracted from the experimental data. The amount of food consumed (measured as volume change in the capillaries) was tracked every 24 h over 6 consecutive days, and capillary tubes were daily replaced with new ones containing fresh solutions. Between 14 and 25 flies were tested per genotype for each food treatment.

### Dietary treatments for evaluation of food consumption

We used 9 dietary conditions, providing flies with one of three concentrations (3, 6, and 12%) each of sucrose and of yeast (the only source of protein offered, but also containing vitamins, minerals, and carbohydrates), provided in separate capillaries. Rather than being mixed together, yeast and sucrose solutions were provided separately to allow flies to mix their intake to meet their particular physiological demands. This means that the flies were able to compensate for dilution of one macronutrient source (yeast or sugar) without having to simultaneously over-eat the other. The term “diet” in our study represents the concentration of nutrients provided in the test solutions, rather than actual intakes of these nutrients. For example, “diet” with 3% sucrose and 3% yeast or yeast autolysate (3S-3Y) does not mean that flies ingested equal amounts of yeast and sucrose. Similarly, “diet” 12S-12Y does not imply consumption of greater amounts of yeast and sucrose than on “diet” 3S-3Y. To lessen this confusion, we mark a dietary treatment as, e.g., 3S-3Y instead of 3S:3Y, which implies an ingested ratio.

To analyze the impact of dietary treatments on metabolism, we recalculated the volumes of ingested yeast and sucrose solutions into actual intakes of carbohydrate and protein. Based on earlier studies, we used values for yeast autolysate composition of 24% carbohydrate and 45% protein (Lee et al., 2008). To calculate the amounts of carbohydrate and protein eaten, the volumes consumed were multiplied by the concentrations of solutions (30 mg/ml for 3%, 60 mg/ml for 6% and 120 mg/ml for 12% solutions).

### Dietary treatments for individuals in which metabolites were determined

It was technically difficult to analyze metabolite levels in single flies from the food consumption experiments described above. Therefore, another set of experiments was performed and these flies were used for measurement of metabolic parameters such as carbohydrate and triacylglyceride contents (assays are described below). Each sample contained about 20 flies that were kept in 162-ml polystyrene vials. The agar (1%) containing yeast autolysate was poured into the bottle cap attached to the bottom of the vial. The space around the cap was filled with sucrose-containing agar. About 900 flies (9 diets × 5 lines × 20 flies) were used for each independent repeat of the experiment.

### Metabolic parameters

Hemolymph was collected between 2 and 3 p.m. Before collection of hemolymph, flies were kept out of food during 1.5 h in order to excrete gut contents. After this time, flies were immediately frozen in liquid nitrogen. Ten frozen flies were weighed, and decapitated by a rigorous agitation in empty Eppendorf tube. Then the fly bodies and heads were mixed with 10 mM sodium phosphate buffer (SPB, pH 7.4) at a 1:5 ratio (weight of flies per volume of the buffer) and centrifuged (3,000×g, 6 min, 4°C). Supernatants were used for determination of hemolymph glucose and trehalose whereas pellet of bodies and heads was used for determination of these carbohydrates along with glycogen and triacylglycerides. To avoid any enzymatic conversion of glucose and trehalose, we inactivated all proteins by heating the supernatants at 70°C for 8 min. After heating, supernatants were re-centrifuged (13,000×g, 15 min, 4°C) in order to pellet heat-denatured proteins.

Bodies and heads remaining after hemolymph collection were homogenized in 10 mM SPB at a ratio 1:10 (weight/volume), centrifuged (13,000×g, 15 min, 4°C), and used for determination of glucose and glycogen levels in the body. The supernatants were heat-denatured as described above. Measurements were performed using a glucose assay kit (Liquick Cor-Glucose diagnostic kit, Cormay, Poland, Cat. #2-203). Trehalose and glycogen were converted into glucose by incubation of supernatants with 7.4 μU/μl porcine kidney trehalase (Sigma-Aldrich Chemie GmbH, #8778) or 1.5 mU/μl (final concentration) amyloglucosidase from *Aspergillus niger* (Sigma-Aldrich Chemie GmbH, #10115) during 18 h at 37°C, respectively.

For triacylgyceride (TAG) determination, six flies were weighed and homogenized in 20 mM phosphate buffered saline (pH 7.4) containing 0.05% Triton X-100 at a ratio 1:34 (weight/volume), boiled, and centrifuged (13,000×g, 10 min) (Rovenko et al., 2015a,b). Triacylglyceride content was measured in supernatants, using Liquick Cor-TG diagnostic kit (Cormay, Poland, Cat. #2-254). Flies of all genotypes were tested in 4–6 independent replicates (Rovenko et al., 2015a).

Total protein content in whole body was measured using Coomassie brilliant blue G-250 dye according to Bradford method. Serum bovine albumin was used as a standard. Data are expressed as mg of protein per g of wet fly body weight (mg/gww).

### Data visualization and statistics

Statistical comparisons were performed using software GraphPad Prism 6 and additionally re-assessed in R v.3.3.1. Analysis of variance (ANOVA) followed by Tukey's test for multiple comparisons with Bonferroni correction was performed for all data. We compared values for either the different fruit fly lines subjected to one particular dietary treatment or between values for one particular line but fed on different dietary treatments. In the latter case, our ANOVA model included the influence of dietary yeast or sucrose alone, or the interaction between both. Boxplots show median values (line inside box) with upper and lower quartiles (bottom and top borders of the box) for 75 and 25% of the data, respectively. Upper and lower whiskers represent 90 and 10% of the data, whereas values outside of the whiskers (outliers) are shown as dots. In the Results section, we present only significant differences between values, except where it is clearly stated that the difference was not significant.

Responses for metabolic variables were mapped onto bi-coordinate intakes of protein and carbohydrate, visualized using thin-plate splines and supported statistically using generalized additive models implemented in R (Solon-Biet et al., 2014).

## Results

### DILPs affect protein rather than sucrose consumption

**Figure 1:**
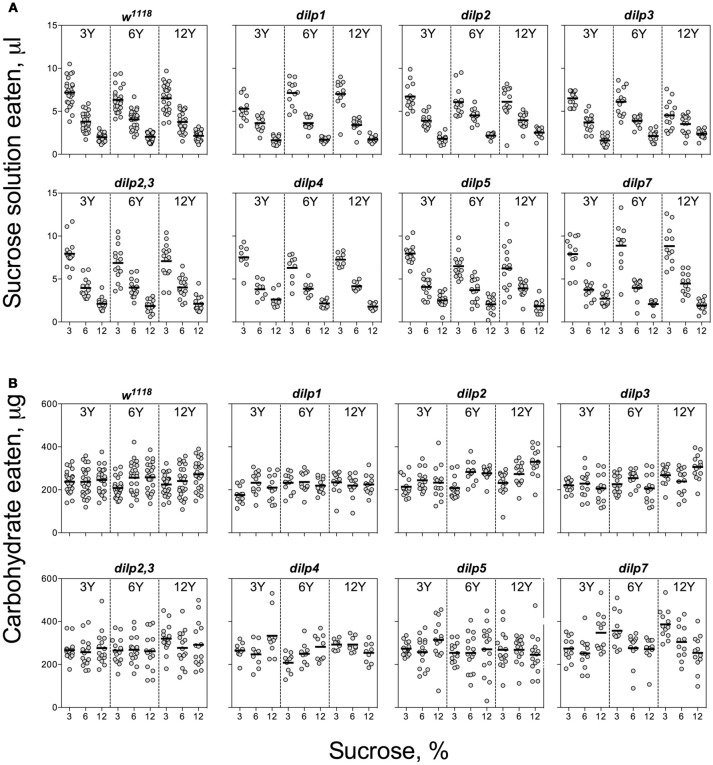
Consumption of carbohydrates by wild type and DILP-deficient fruit flies. **(A)** Volumes of sucrose solution ingested. **(B)** Amounts of carbohydrate consumed. Tubes with tested fruit flies were supplied by pair of capillaries: one filled with solution of sucrose, and the other filled with solution of yeast autolysate. It was allowed flies to choose between capillaries for eating either yeast autolysate (mainly, source proteins and essential micronutrients such as vitamins, nitrogen bases, etc.) or sucrose (a source of carbohydrates). The combinations of 3, 6, and 12% sucrose, and 3, 6, and 12% of yeast autolysate were used. Data are *n* = 3 replicates (each containing 3–8 flies) for each diet and fly line combination.

Individuals of the wild type line, *w*^1118^, and the mutant lines consumed larger volumes of solutions with low than high sucrose concentrations, indicative of compensatory feeding for sugar. Hence, wild type flies showed a decrease in consumption of sucrose solution when the concentration of carbohydrate was increased from 3 to 12% [*F*_(1, 221)_ = 438, *p* < 0.001; Figure 1A, Datasheets S2, S3]. All the mutant lines showed similar trends, consuming lower volumes of solutions with high carbohydrate concentrations. However, *dilp5* and *dilp7* mutants ingested 25 and 30% more sucrose on 12S-3Y diet than the wild type, respectively (Datasheet S4). Flies of *dilp2* mutant line ate 14% more sucrose solution when given 12% solutions. Interactions of diet-dependent consumption of sucrose solution were observed between *dilp1* and *dilp7*, and *dilp3* and *dilp7* mutants (Tables S5, S6).

**Figure 2:**
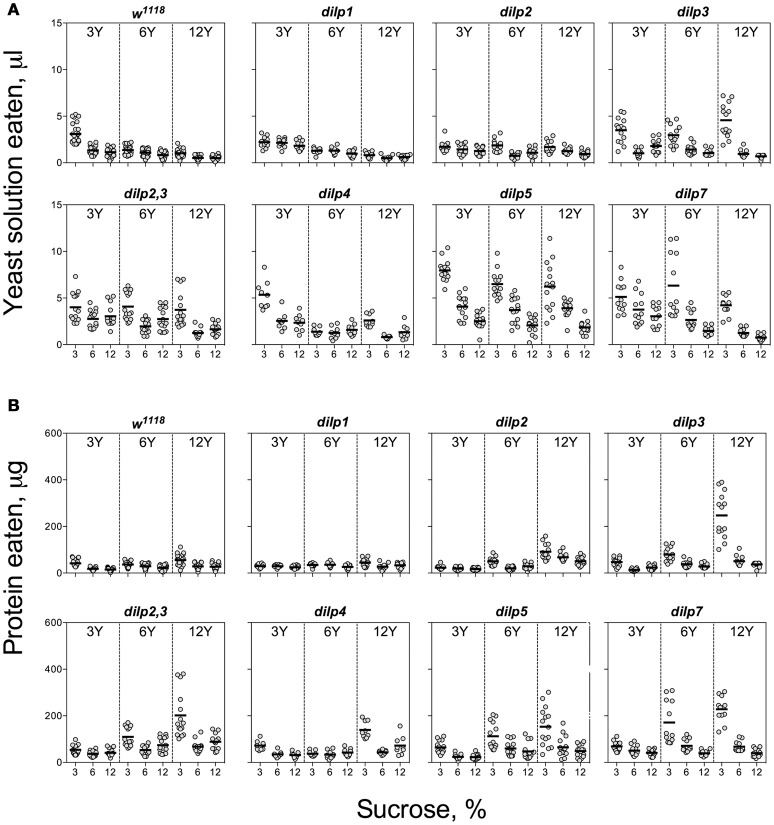
Consumption of protein-rich food source by control and DILP-deficient fruit flies. **(A)** Volumes of yeast autolysate solution ingested. **(B)** Amounts of protein consumed. Data are *n* = 3 replicates (each containing 3–8 flies) for each diet and fly line combination.

The wild type flies demonstrated compensatory feeding for protein, although less marked than for carbohydrate, and consumed the biggest volume of yeast autolysate solution (Y) at its low concentrations (3 and 6%) when these were paired with a low concentration of sucrose (3%) (Figure 2A). Mutations of *dilp1* and *dilp2* abrogated increased consumption of Y on the 3S−3Y diet. Also, *dilp2* mutants consumed 28% less volume than the wild type on 6S-6Y diet. The wild type flies consumed 1.5- to 2.2-fold more Y on diets with 3% sucrose than with 12% sucrose; comparable values for *dilp*2 mutants were 1.4- to 1.7-fold. The *dilp2* mutants also consumed 1.4- to 2.1-fold more Y than the wild type on diets with 12% Y (Datasheet S4). Flies of *dilp2,3* and *dilp7* lines ate 1.3- to 4.3-fold more Y on all diets as compared to the wild type flies. Similarly, *dilp4* and *dilp5* mutants ingested 1.2- to 2.7-fold more Y than the wild type on all diets, except for *dilp4* mutants on 3S-6Y and 6S-6Y diets and *dilp5* mutants on 6S-3Y diet.

Despite clear evidence for compensatory feeding for carbohydrate (above), the calculated amount of carbohydrate consumed was not maintained constant across food treatments and was dependent on sucrose concentration for the wild type flies [*F*_(1, 221)_ = 11.5, *p* < 0.001], being higher on the high as compared to the low sucrose solutions (Figure 1B). Dependence of the amount of consumed carbohydrate on sucrose concentration was also seen for *dilp2, dilp3, dilp4, dilp5*, and *dilp7* lines (Table S5). However, carbohydrate consumed by the *dilp2,3* mutants was dependent on the concentration of yeast provided [*F*_(1, 131)_ = 4.72, *p* < 0.05]. The *dilp2*,*3, dilp4*, and *dilp7* mutant lines ingested 21, 26, and 58% more carbohydrates on diet 3S-12Y than the *w*^1118^ flies, respectively. Flies of *dilp2,3, dilp5*, and *dilp7* mutant lines also consumed 1.3-, 1.2-, and 1.6-fold more carbohydrates than the wild type on 3S-6Y diet. In addition, the *dilp4, dilp5*, and *dilp7* mutants increased carbohydrate consumption by 25–33% on the 12S-3Y diet. The *dilp4* and *dilp7* mutants also ate 13 and 19% more carbohydrates on the 6S-12Y diet as compared to *w*^1118^ flies, respectively. Somewhat higher carbohydrate consumption (15 and 21%) was also observed for *dilp5* flies on 3S-3Y and 3S-6Y diets.

The pattern for amount of protein consumed followed the concentration of Y provided. The *dilp2,3* and *dilp7* mutants consumed 1.3- to 4.3-fold more protein on all diets as compared to the wild type (Datasheet S4). Mutants on *dilp2, dilp2,3, dilp3, dilp4, dilp5*, and *dilp7* also ingested 1.4-, 3.0-, 4.3-, 2.3-, 2.7-, and 4.3-fold greater amounts of protein on 3S-12Y diet than the wild type flies, respectively (Figure 2B). Similarly, *dilp2, dilp3, dilp2,3, dilp5*, and *dilp7* mutants demonstrated 1.3-, 2.0-, 2.5-, 2.1-, and 3.3-fold higher amounts of ingested protein on 3S−6Y diet as compared to the wild type.

**Figure 3:**
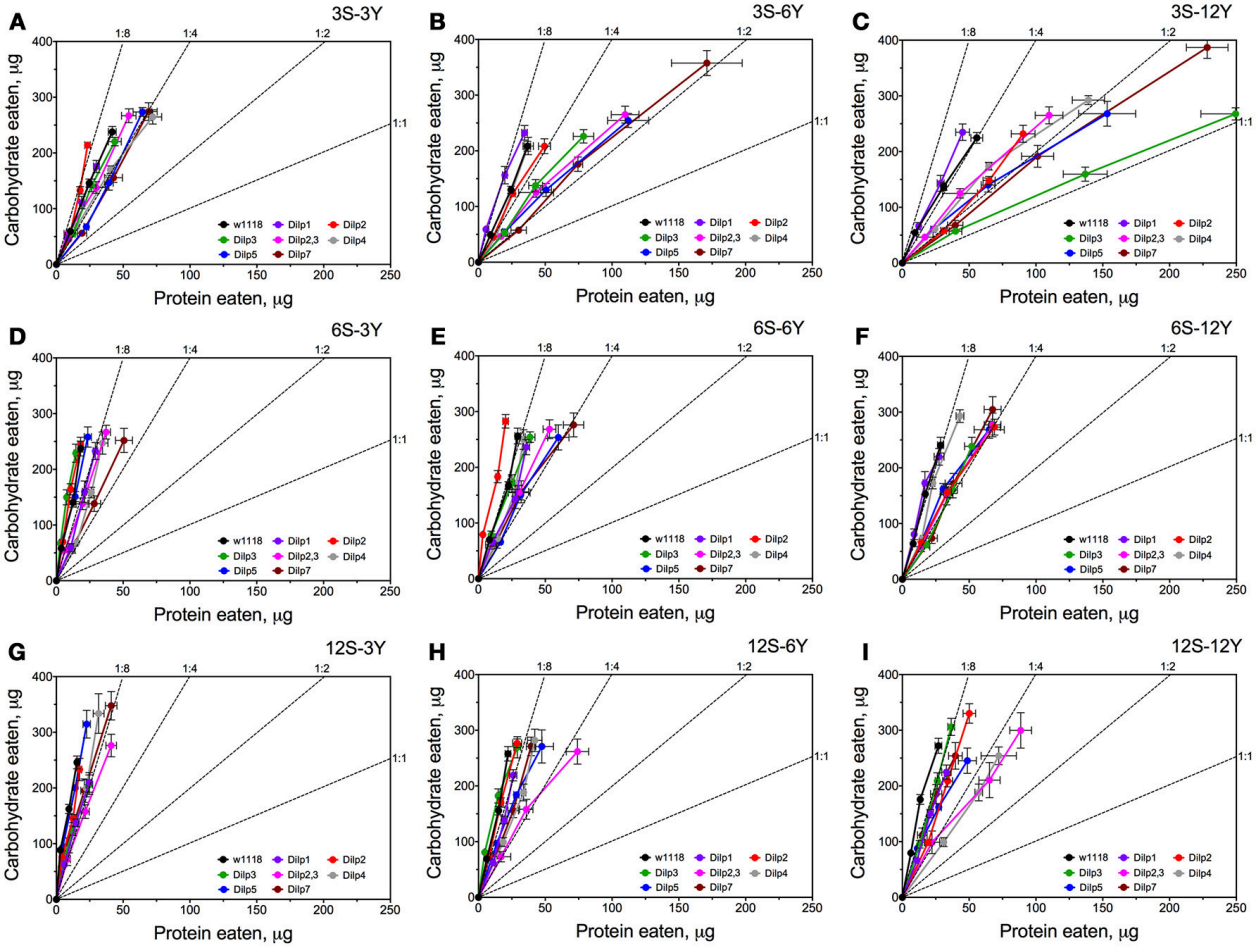
Nutrient intake trajectories resulting from volumes ingested of the two food solutions (sucrose and yeast) for all fruit fly lines tested. Data are *n* = 3 replicates (each containing 3–8 flies) for each diet and fly line combination. Panels **(A–I)** correspond to dietary treatments 3S-3Y, 3S-6Y, 3S-12Y, 6S-3Y, 6S-6Y, 6S-12Y, 12S-3Y, 12S-6Y, and 12S-12Y.

Knowing the volumes of the different solutions consumed and the concentrations of nutrient within the solutions, we were able to plot the relationships between protein and carbohydrate eaten using nutritional geometry (Figure 3). Small differences between fly lines were observed on 3S-3Y and 12S-3Y diets. However, flies tended to eat more carbohydrate than protein, keeping the protein:carbohydrate (P:C) ratio of ingested nutrients as 1:8 or 1:4. On the 3S-3Y diet, the *dilp2* mutant maintained a P:C ratio of 1:8, whereas *dilp1, dilp4*, and *dilp7* mutants stuck to 1:4 ratio. The other lines, including wild type, showed a P:C ratio between 1:8 and 1:4. On the 12S-3Y diet, trajectories for all lines converged close to a 1:8 ratio. On the 3S-12Y diet, both the wild type and *dilp1* mutant converged on P:C ratio of 1:4. However, P:C trajectories for *dilp2, dilp2,3, dilp4, dilp5* and *dilp7* mutants on this diet were skewed toward a ratio of 1:2, whereas *dilp3* mutant flies ingested even more protein and less carbohydrate, reaching a 1:1 ratio. Of note, flies of the *dilp7* line consumed the largest amount of carbohydrate among all lines on the 3S-12Y diet and rather large amounts of protein. However, high sugar concentration of the 12S-12Y diet leveled the effect observed for mutant lines on the 3S-12Y diet. These observations indicated that whereas wild type flies on 3S-12Y diet restricted sipping from the autolysate-containing capillary, the mutant lines sipped equally from both capillaries.

### Different DILPs influence hemolymph glucose in a diet-dependent manner

**Figure 4:**
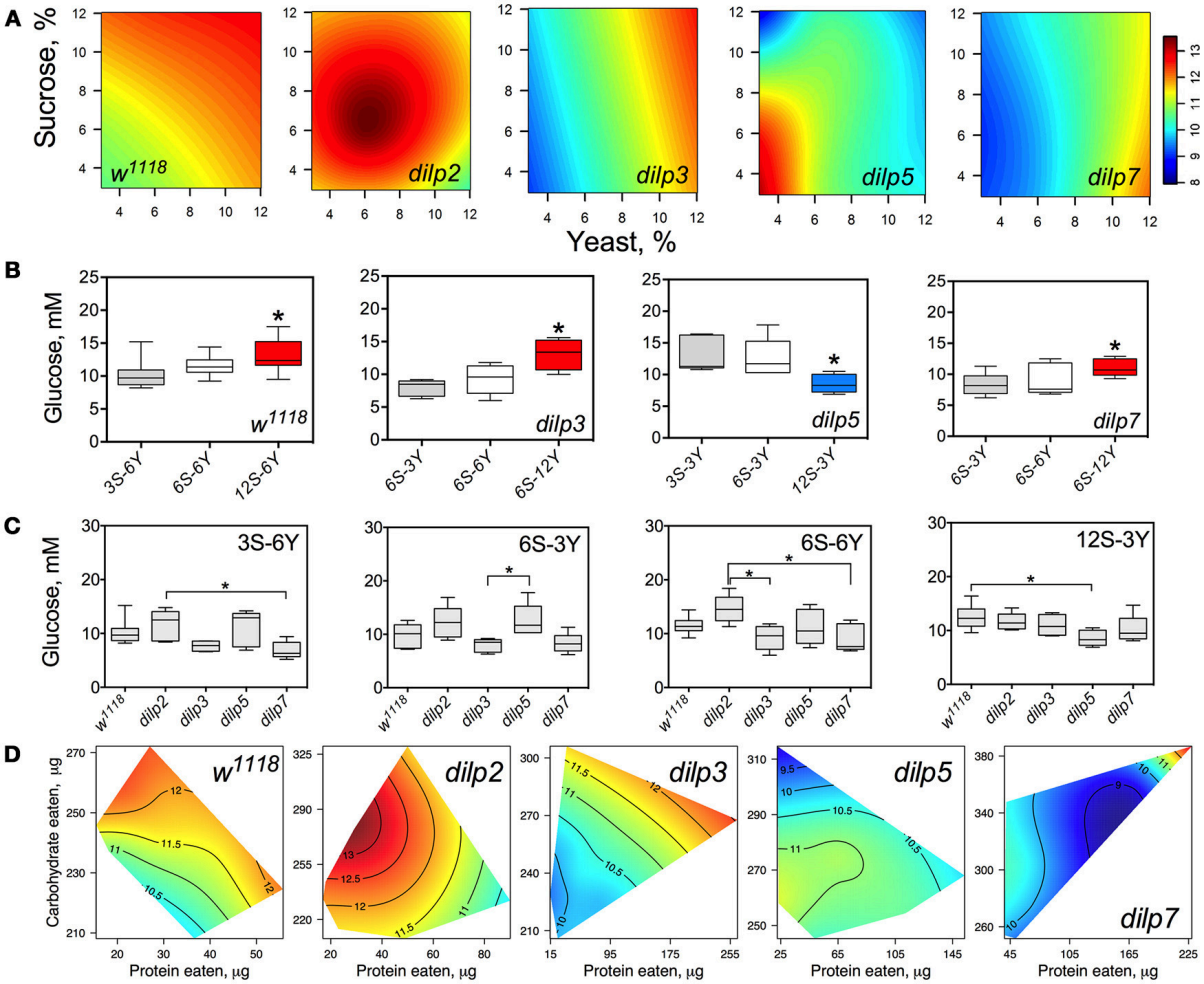
dILPs deficiency changes the diet-dependent pattern of glucose concentration in fruit fly hemolymph. **(A)** Dietary response surfaces depicting dependence of concentration of glucose in fly hemolymph (mM) on concentration of yeast and sucrose in the diet. There were tested nine combinations of sucrose (3, 6, and 12%) and yeast (3, 6, and 12%). In each case, the flies of tested lines were able to choose between of sucrose- or yeast-containing medium. All five surfaces are placed under one scale. **(B)** The remarkable differences between hemolymph glucose values in flies of each line kept on different diets. Red-colored boxes designate statistically significant increase (indicated by the asterisk; *p* < 0.05, Tukey's test with Bonferroni correction) whereas blue-colored boxes designate statistically significant decrease as compared to the values for flies consumed diet designated by gray color box. **(C)** The remarkable differences between hemolymph glucose values in flies of different lines on particular diets. **(D)** Dietary responses surface depicting dependence of hemolymph glucose in wild type and *dilp* mutant lines on amounts of protein and carbohydrate consumed. Each surface has own scale shown by contour lines. Data are *n* = 3–6 replicates for each diet and fly line combination.

Hemolymph glucose (HG) in wild type flies was affected by diet and was dependent on dietary sucrose (*p* = 0.026) rather than yeast (*p* = 0.601) or their interaction (*p* = 0.130) (Figure 4A; Table S7). Lack of DILPs reshapes the relationship between HG and dietary components. Particularly, in *dilp3* and *dilp7* mutant flies HG was dependent on dietary yeast (*p* = 0.001 and 0.012, respectively). Neither yeast nor sucrose affected HG in *dilp2* and *dilp5* mutant flies.

The wild type flies showed 27% higher HG on 12S-6Y diet than those fed 3S-6Y diet (Figure 4B). HG was 57 and 31% higher on the 6S-12Y diet compared with 6S-3Y in *dilp*3 and *dilp*7 mutants, respectively. However, *dilp5* mutants had by 36% lower HG when fed 12S-3Y diet compared to 3S-3Y. The differences in HG in flies of different genotypes were observed on distinct diets (Figure 4C). Particularly, *dilp7* mutant flies had 14 and 20% lower HG on diets 3S-6Y and 6S-6Y in comparison with values for *dilp2* mutant flies. In addition, HG was lower by 35% in *dilp3* mutants compared to *dilp2* on 6S-6Y diet. The *dilp5* mutants had 32% higher HG than *dilp3* flies fed 6S-3Y diet. Loss of *dilp5* significantly decreased HG on high-sucrose low-yeast diet 12S-3Y.

Loss of DILPs affected HG in response to protein and carbohydrate intake (Figure 4D). The highest HG in wild type flies was observed for high-carbohydrate and low protein intake. In *dilp2* mutant flies, HG was maximized with consumption of moderate amounts of sucrose and low amounts of protein. Maximum values for HG were observed in *dilp3* mutants that consumed high amounts of protein and moderate amounts of carbohydrate. Oppositely, the highest HG values in *dilp5* mutants were observed with the consumption of moderate amounts of carbohydrate and were independent of the amount of protein consumed. Mutation on *dilp7* resulted in highest HG under high protein and high carbohydrate intakes.

### DILP3 is involved in regulation of hemolymph trehalose on low-protein and high-carbohydrate diet

**Figure 5:**
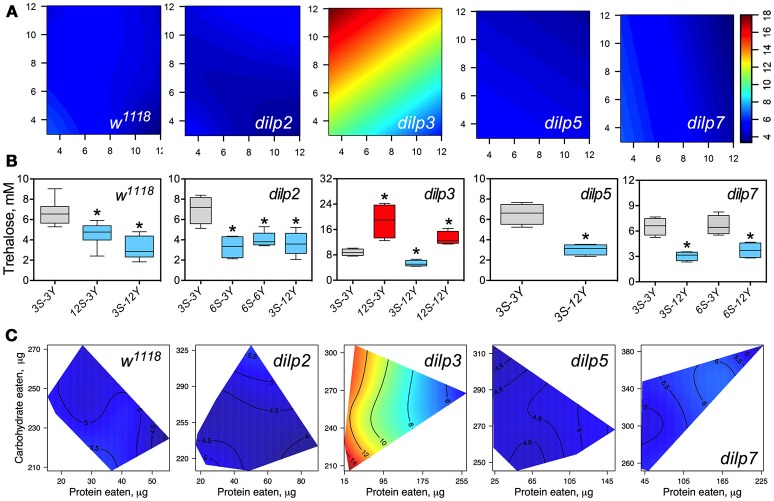
The effect of mutations on *dilp* genes on the diet-dependent pattern of trehalose concentration in fruit fly hemolymph. **(A)** Dietary response surfaces representing dependence of concentration of trehalose in fly hemolymph (mM) on concentrations of yeast and sucrose in the diet. There were tested nine combinations of sucrose (3, 6, and 12%) and yeast (3, 6, and 12%). Axes' titles are the same as in Figure 4. In each case, the flies of tested lines were able to choose between of sucrose- or yeast-containing medium. All five surfaces are placed under one scale. **(B)** The remarkable differences between hemolymph trehalose values in flies of each line kept on different diets. Red-colored boxes designate statistically significant increase (indicated by the asterisk; *p* < 0.05, Tukey's test with Bonferroni correction) whereas blue-colored boxes designate statistically significant decrease as compared to the values for flies diet treatment 3S-3Y designated by gray color box. **(C)** Dietary response surfaces showing dependence of hemolymph glucose in wild type and *dilp* mutant lines on amounts of protein and carbohydrate consumed. Each surface has own scale shown by contour lines. Data are *n* = 3–6 replicates for each diet and fly line combination.

The concentration of trehalose in hemolymph (HT) of wild type flies was dependent on the concentration of dietary yeast (*p* = 0.001) and was modulated by sucrose (*p* = 0.013 for the interaction between yeast and sucrose; Figure 5A; Table S8), although sucrose concentration as a main effect did not influence HT (*p* = 0.557). HT in *dilp2* mutants was dependent on sucrose (*p* = 0.002). Whereas, there was no main effect of yeast (*p* = 0.9517), there was a statistically significant interaction between sucrose and yeast concentration (*p* = 0.005) on trehalose levels. The concentration of hemolymph trehalose in *dilp3* mutants was strongly dependent on dietary yeast (*p* = 0.001) and sucrose (*p* < 0.001). A weak dependence of HT on dietary sucrose was found for the *dilp5* mutant line (*p* = 0.049). Indeed, the HT values of *dilp5* mutants were very similar in flies kept on all diets. There was also a dependence of HT on both nutrients (*p* = 0.032) in *dilp5* mutants. A strong dependence of HT on the concentration of dietary yeast was found for the *dilp7* mutant (*p* < 0.001).

Wild type flies fed 3S-12Y diet had 2.3-fold lower HT values than those kept on 3S-3Y diet (Figure 5B). We also observed 27 and 32% lower HT values in the wild type flies fed on 12S-3Y and 12S-12Y diets, respectively, when compared to 3S-3Y diet (Figure 5B). The *dilp2* flies kept on 3S-3Y diet had 2.1-, 1.9-, and 2.0-fold higher concentration of hemolymph trehalose than flies fed on 6S-3Y, 6S-6Y, and 3S-12Y diets, respectively. In the *dilp*3 line, the lowest HT value was observed on the 3S-12Y diet, being 42% lower compared to that on 3S-3Y diet. The *dilp3* flies fed 12S-3Y diet had 2.2-fold higher trehalose concentration in hemolymph than those fed 3S-3Y diet, whereas those fed 12S-12Y diet showed 1.4- and 2.5-fold higher HT than the flies on 3S-3Y and 3S-12Y diets, respectively. Only *dilp5* mutant flies that consumed 12S-3Y diet had lower (29%) hemolymph trehalose than flies on 3S-3Y diet. The *dilp7* flies on 3S-12Y and 6S-12Y diets contained 2.1- and 1.7-fold less trehalose in hemolymph than on 3S-3Y and 6S-3Y diets, respectively.

Interestingly, *dilp3* mutant flies had 1.6- to 4.0-fold higher HT values compared to the wild type line (Figures 5A,C). The smallest difference between the lines was observed on the 6S-6Y diet, while the largest differences occurred on 12S-3Y diet. Trehalose concentration in hemolymph was not significantly different between *w*^1118^ and *dilp5* or *dilp7* flies, whereas flies of the *dilp2* line had 41% lower hemolymph trehalose concentration on 6S-3Y diet than *w*^1118^ flies.

A clear dependence of HT concentration on the amounts of macronutrients eaten was seen only for *dilp3* and *dilp7* mutants (Figure 5C). Particularly, *dilp3* mutant flies had the highest concentration HT values only when consumed low amounts of protein; HT in *dilp3* flies was only marginally influenced by the amount of carbohydrate consumed. The opposite situation was observed for the *dilp7* mutant. The peak in concentration of circulating trehalose was observed when high amounts of carbohydrate and protein consumed. At the same time, the lowest values of circulating trehalose were observed when low amounts of protein and carbohydrate were eaten.

### DILP2 and DILP5 are involved in regulation of glycogen accumulation independently of diet

**Figure 6:**
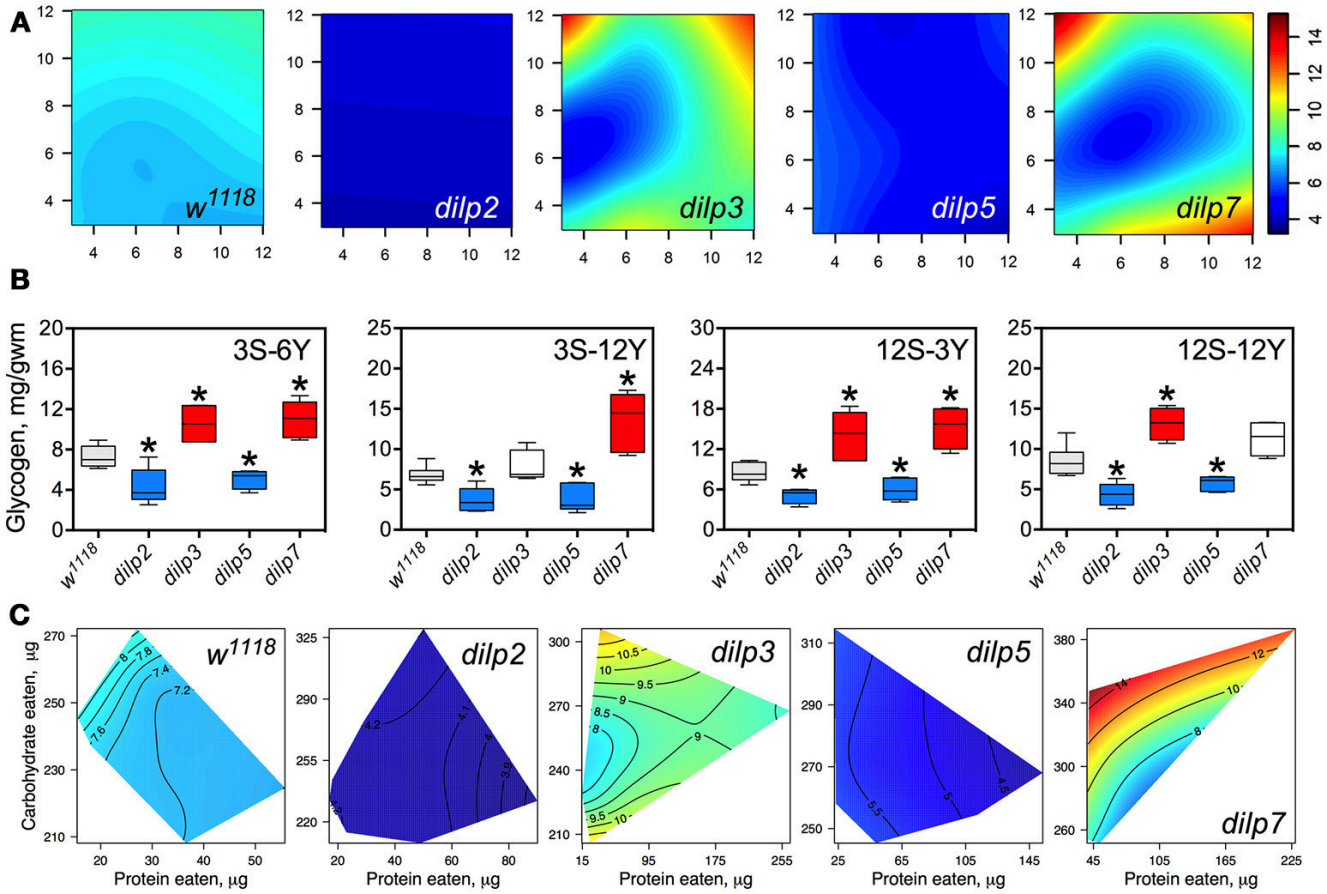
The effect of *dilp* mutations on the diet-dependent pattern of glycogen accumulation in fruit fly body. **(A)** Dietary response surfaces depicting dependence of glycogen content in the body of individuals of wild type (*w*^1118^) and *dilp* mutant lines on concentrations of yeast and sucrose in the diet. Axes' titles are the same as in Figure 4. **(B)** The remarkable differences in glycogen content between investigated fruit fly lines. Red-colored boxes designate statistically significant increase (indicated by the asterisk; *p* < 0.05, Tukey's test with Bonferroni correction) whereas blue-colored boxes designate statistically significant decrease as compared to the values for the wild type flies. **(C)** Dietary response surfaces showing dependence of glycogen content in wild type and *dilp* mutant lines on amounts of protein and carbohydrate consumed. Each surface has own scale shown by contour lines. Data are *n* = 3–6 replicates for each diet and fly line combination.

Glycogen content of fly bodies was dependent on sucrose concentration in wild type flies (*p* = 0.002; Figure 6A; Table S9) and in *dilp3* and *dilp7* flies. Glycogen content in the *dilp*7 mutant resembled the diet-dependent pattern attributed to the *dilp3* mutant. However, glycogen accumulation in *dilp5* and *dilp7* mutants was dependent on both yeast and sucrose (*p* = 0.031 and *p* = 0.004, respectively), and was also affected by dietary yeast in the *dilp5* mutant (*p* = 0.027; Table S9). However, glycogen content of *dilp2* mutants was not dependent on either dietary sucrose, or yeast, or their combination.

The *dilp2* mutants showed 1.5- to 2.4-fold lower glycogen content on all diets than the wild type line (Figure 6A). The smallest difference between these lines was observed on the 12S-3Y diet, while the largest difference was on the 12S-6Y diet (Figure 6B). The mutant *dilp*3 line accumulated larger amounts of glycogen on diets 3S-6Y, 12S-3Y, and 12S-12Y than the wild type. The *dilp5* mutant had 1.3- to 2.2-fold lower glycogen content than the wild type on high sugar (12S) and high yeast diets (12Y) (Figure 6B). The biggest difference between these lines was observed on the 3S-12Y diet. The *dilp7* mutant accumulated higher glycogen than the wild type on the 3S-6Y, 3S-12Y, and 12S-3Y diets.

Significantly, 1.9-fold higher glycogen content was observed on the 12S-12Y diet compared to 3S-12Y in *dilp3* flies, whereas *dilp7* flies accumulated 2.0-fold more glycogen on 12S-3Y diet compared to 3S-3Y diet.

The amount of stored glycogen increased with increasing in carbohydrate consumption in wild type flies (Figure 6C) and deficiency in particular DILPs changed this pattern. In *dilp3* mutants, the dependence of glycogen accumulation on the amount of carbohydrate consumed was disrupted, being maximal at either very low or very high amounts of carbohydrates eaten. In *dilp5* mutants, the amount of glycogen decreased with the amount of protein eaten but did not depend on the amount of carbohydrate eaten. Consumption of carbohydrates promoted glycogen accumulation in *dilp7* mutants but protein consumption lowered glycogen accumulation.

### DILP3, DILP5, and DILP7 affect accumulation of triacylglycerides

**Figure 7:**
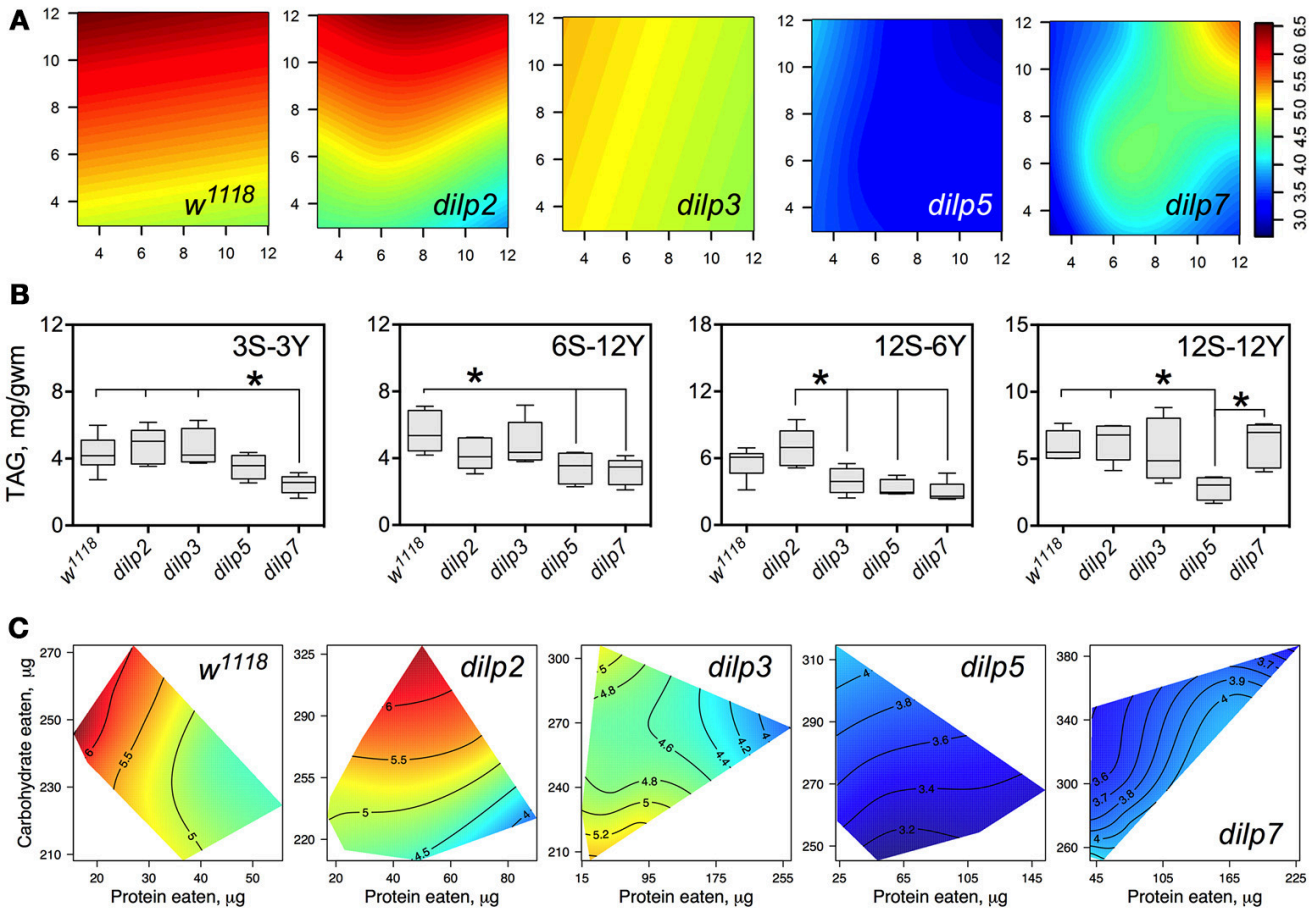
The effect of *dilp* mutations on the diet-dependent pattern of triacylglyceride (TAG) accumulation in fruit fly body. **(A)** Dietary response surfaces depicting dependence of TAG content in the body of individuals of wild type (*w*^1118^) and *dilp* mutant lines on concentrations of yeast and sucrose in the diet. Axes' titles are the same as in Figure 4. **(B)** The remarkable differences in TAG content between investigated fruit fly lines. **(C)** Dietary response surfaces showing dependence of TAG content in wild type and *dilp* mutant lines on amounts of protein and carbohydrate consumed. Each surface has own scale shown by contour lines. Data are *n* = 3–6 replicates for each diet and fly line combination.

The wild type flies and *dilp2* mutant flies accumulated high amounts of triacylglycerides (TAG) in their bodies on high carbohydrate diets, strongly linked to dietary sucrose (*p* = 0.0003 and *p* = 0.0001, respectively; Figure 7A; Table S10). Content of TAG in *dilp2* mutants was also dependent on dietary yeast (*p* = 0.0123). A similar effect characterized *dilp7* flies, in which TAG content was also affected by both nutrients (*p* = 0.0194 and 0.0168 for sucrose and yeast, respectively).

The wild type flies had 74% more TAG on 12S-3Y diet compared to 3S-3Y diet. In addition, these flies had 21% more TAG on 12S-12Y diet compared to 3S-12Y. The difference was less pronounced on diets 3S-6Y and 12S-6Y. The *dilp2*-deficient flies showed a pattern of diet-dependent TAG accumulation similar to the wild type. However, differences in TAG content between low and high sugar diets were more marked than in the wild type, having been shifted more to the high yeast diets. In particular, this difference was 2.4-fold between 3S-12Y and 12S-12Y diets, and 1.7-fold between 3S-6Y and 12S-6Y diets.

Mutations on *dilp3, dilp5*, and *dilp7* changed the pattern of TAG accumulation on high sugar diets. Flies of the *dilp5* line had 1.5- to 2.3-fold lower amounts of TAG in their bodies compared to the wild type on all high sucrose diets, and on diets 6S-6Y, 6S-12Y, and 3S-6Y (Figure 7B). The *dilp3* mutants showed 1.6- and 1.7-fold lower amounts of TAG than the wild type and *dilp2* mutant on 12S-6Y, respectively. The *dilp7* mutant had 1.5- to 1.9-fold lower TAG content than *w*^1118^ flies on all low yeast diets. A 1.4-, 1.5-, and 2.4-lower body TAG content compared to the wild type was observed on 3S-12Y, 6S-12Y, and 12S-6Y diets, respectively.

In many cases, flies reared on diets with high carbohydrate content accumulated the highest amounts of TAG (Figure 7C). When we assessed dependence of TAG content on amounts of carbohydrate and protein eaten, we found a clear decrease of TAG content in fly bodies when high amounts of protein were consumed in both wild type flies and *dilp2* mutants.

### Mutations on *dilp3* and *dilp7* lead to increased body glucose on high-yeast diets

The concentrations of dietary sucrose and yeast had a relatively weak influence on the concentration of glucose in fly bodies, and this was observed for all lines. A dependence on sucrose concentration was exhibited by the wild type and *dilp7* lines (*p* = 0.041 and 0.0064, respectively; Figure S1A, Table S11). A dependence of body glucose on yeast concentration was observed for the *dilp3* (*p* = 0.018) and *dilp7* mutants (*p* = 0.010).

The wild type flies had 34% higher levels of body glucose on 12S-12Y diet as compared to 12S-3Y (Figure S1B). Similarly, *dilp7* flies accumulated 41% more glucose on the 12S-12Y diet than on 12S-3Y diet. The *dilp3* mutant flies showed significant differences in this parameter only when fed on low-sugar-low-yeast diets. The *dilp2* and *dilp5* mutants had 25 and 21% lower levels of glucose in their bodies on the 3S-6Y diet vs. wild type flies, respectively. Interestingly, *dilp3* and *dilp7* mutant flies had 1.3- and 1.8-fold higher levels of body glucose on all diets with 12% sucrose and on 3S-6Y and 3S-12Y diets compared to the wild type flies. Additionally, *dilp3* mutants had a 1.6-fold higher level of body glucose on the 6S-3Y diet.

The concentration of glucose in fly bodies reached the highest value on high intakes of protein in the wild type line and was dependent on amount of consumed protein in general (Figure S1B). A somewhat similar situation was observed for the *dilp3* line. However, the transition from low to high values of body glucose was steeper in *dilp3* flies than in *w*^1118^ flies. The highest values were observed at high levels of both consumed protein and carbohydrate. A similar pattern was also seen for the *dilp7* mutant, however the lowest value was observed at moderate intakes of protein, unlike *w*^1118^ and *dilp3* flies where the lowest value of body glucose corresponded the lowest intakes of protein.

### All *dilp* mutations lead to specific body trehalose response to diet

Body trehalose levels were slightly affected by diet in wild type flies. The lowest value was observed on the 6S-12Y diet. The highest amount of body trehalose was observed on 3S-12Y and 12S-3Y diets. However, mutation of *dilp2* suppressed these diet-dependent responses and led to near equal accumulation of trehalose in the bodies of these flies independently of diet (Table S12). Mutation on *dilp3* led to a relatively small decrease in levels of body trehalose on diets with low concentrations of yeast extract. However, mutation of *dilp5* caused a large decrease in body trehalose levels on diets with either low (3%) or high (12%) concentrations of sucrose (Figure S2). Mutants on *dilp7* exhibited similar patterns of body trehalose as compared to the wild type line but with a more pronounced increase of body trehalose on 3S-3Y, 3S-6Y, and 3S-12Y diets.

### Lack of particular DILPs did not affect the influence of diet on body mass

Body mass was dependent on dietary sucrose in wild type flies (*p* = 0.022) with a marginally statistically significant effect of dietary yeast (*p* = 0.049) (Table S13). The *dilp2* and *dilp5* mutants had about 14–15% lower body mass on 12S-6Y diet (Datasheet S4). However, there were no significant effects of diet on body mass in other *dilp* mutants.

## Discussion

### Food consumption and appetite

As in previous studies, we have found that fruit flies consume more food on diets with low rather than higher percentages of carbohydrates, indicative of compensatory feeding for carbohydrates (Lushchak et al., 2011, 2012, 2014, 2016; Rovenko et al., 2015a). DILPs are known to be connected with signaling pathways that control appetite (Wu et al., 2005; Itskov and Ribeiro, 2013; Nässel et al., 2013; Luo et al., 2014; Lushchak et al., 2015), and diminution of insulin signaling leads to increased consumption of unpalatable food by fruit flies or their larvae (Wu et al., 2005; Luo et al., 2014). In the present study, the lack of a functional DILP4, DILP5, or DILP7 led to significantly increased food consumption on almost all diets. However, the lack of either DILP2 or DILP3 led to increased food consumption only on high protein diets. Notably, *dilp7* mutants had the most pronounced increase in food intake on low sucrose concentrations. These mutants also showed compensatory responses to changes in concentration of the yeast solution, for example consuming a substantially greater volume of food than wild type flies on the 3S-3Y and the 3S-6Y diets. DILP7 is believed to be a relaxin-like peptide in *Drosophila*, regulating egg laying decisions and thus it would have a major role to play for the female flies used in the present study. It is notable that protein and carbohydrate intake are essential for reproduction, with protein appetite being tightly coupled to mating and egg production (Lee et al., 2008; Simpson and Raubenheimer, 2012; Sisodia and Singh, 2012; Lushchak et al., 2014; Leitão-Gonçalves et al., 2017; Liu et al., 2017). Interestingly, human relaxins have been shown to be orexigenic (McGowan et al., 2008; Grosse et al., 2014). Partial co-expression of *dilp7* with short neuropeptide F is involved in feeding behavior (Nässel et al., 2013) and, additionally, DILP7 has been reported to be involved in the regulation of feeding behavior by other authors (Cognigni et al., 2011). Interestingly, the effect was obtained by inactivation of Dilp7 producing neurons. Increased appetite is also characteristic of other DILP mutants, though to a lesser degree. This may suggest that other DILPs also function at least partially as relaxin-like peptides (Nässel and Vanden Broeck, 2016). However, this poses the question as to whether specific DILPs, especially DILP7, may bind to receptors other than the *Drosophila* insulin receptor (dInR). Human relaxins and insulin-like peptides are known to bind to specific G-protein coupled receptors distinct from the tyrosine kinase type insulin receptor (Bathgate et al., 2013).

It was shown recently that responses to a continuous lack of protein and carbohydrates are mediated by distinct dopaminergic neurons (Liu et al., 2017). Moreover, activation of protein feeding simultaneously inhibited carbohydrate feeding. Our results add complexity to these data, suggesting that the response is additionally mediated by DILPs.

### Glucose and trehalose

The only DILP mutation which led to substantial increase in the level of hemolymph glucose was DILP2. Mutation of all the other DILPs resulted in a slight decrease in hemolymph glucose on some diet treatments and a slight increase on others (e.g., *dilp5* mutant). The regulation of hemolymph glucose levels by DILP2 has also been confirmed by other authors (Zhang et al., 2009), and its physiological proximity to insulin is supported by the similarity of amino acid sequences of the two peptides (Brogiolo et al., 2001). The dependence of hemolymph glucose on protein intake by *dilp3, dilp5*, and *dilp7* mutants suggests that DILPs may also be involved in regulation of distinct branches of glucose metabolism such as glycolysis, gluconeogenesis, glycogenesis, and the pentose phosphate pathway. Particularly, the level of circulating glucose may depend on the balance between cellular influx and efflux of glucose, glycogen synthesis and breakdown, synthesis of glucose from organic or amino acids via gluconeogenesis, as well as on glucose catabolism via glycolysis and/or pentose phosphate pathways. In fruit flies, glucose can also be used for trehalose synthesis. However, circulating trehalose levels showed an inverse relationship to circulating glucose in the *dilp3* mutant (Figure 3C vs. Figure 4D for *dilp3* mutant). This suggests that a lack of DILP3 may result in conversion of glycogen stores into trehalose instead of glucose (Yamada et al., 2018). This possible conversion was inhibited by an increased intake of protein. Therefore, our current data imply a role of DILP3 in regulating trehalose concentration in hemolymph. This finding is not consistent with previous data, which showed that a knockdown of the *dilp2* gene but not *dilp3* gene led to an increase in trehalose content in whole flies (Broughton et al., 2008; Kannan and Fridell, 2013). However, the diet used in that study was more concentrated than most of the diets in the current study. Notably, it has been shown that trehalose promotes selective secretion of DILP3 (Kim and Neufeld, 2015). Interestingly, *dilp3* mutants also had high levels of glucose in their bodies. A similar pattern of diet-dependent accumulation of trehalose in the body was observed for *dilp7* mutants although this accumulation was not reflected in an increase in hemolymph trehalose levels, suggesting that *dilp7* is not involved in regulation of this branch of metabolism.

### Glycogen and triacylglycerides

Using nutritional geometry, we have shown that both *dilp2* and *dilp5* are involved in regulation of glycogen synthesis in fruit flies, a function that has not been reported previously for these DILPs (Broughton et al., 2008; Grönke et al., 2010). Glycogen, along with proteins, contributes to fly weight, what is reflected in our study where *dilp2* and *dilp5* mutants were significantly lighter than controls on a high carbohydrate diet. Earlier, it was shown that *dilp5* expression was dependent on dietary carbohydrate (Morris et al., 2012; Pasco and Léopold, 2012). On the other hand, in a previous study we did not find a significant change in *dilp5* expression with an increase in sugar concentration in the larval diet (Rovenko et al., 2015a). However, *dilp5* expression was dependent on the type of carbohydrate: increased glucose concentration did not change *dilp5* expression, but fructose suppressed steady-state levels of *dilp5* transcripts (Rovenko et al., 2015b).

The current data show that TAG and glycogen accumulation were most pronounced on high sucrose diets in wild type flies. Lack of DILP5 led to a decrease in TAG content for all treatments. Hence, DILP5 seems to be especially necessary for nutrient sensing on high carbohydrate and high yeast diets, and also for converting ingested food into carbohydrate and lipid storage on these diets. Recent data on *dilp5* expression confirm these suggestions. In particular, expression of *dilp5* was dependent on both consumed carbohydrate and protein in the study of Post and Tatar (2016), and was triggered by yeast in larvae in a study by Okamoto and Nishimura (2015).

The role of Dilp5 in regulation of glycogen and TAG synthesis/breakdown has not been reported previously. Our study design was notable in two respects. First, we allowed flies to choose between protein and carbohydrate sources. This choice takes into account the impact of a *dilp* mutation on nutrient sensing. Indeed, *dilp* expression and release of the peptides from neurosecretory cells is dependent on availability of particular nutrients. The sensory responses to particular nutrients which are transduced to insulin-producing cells was shown to be mediated by biogenic amines (Luo et al., 2014) and food odors (Lushchak et al., 2015). Secondly, we created conditions where flies were relatively free to fly and otherwise move around, thereby expending energy and creating a demand for glycogen and TAG catabolism.

As compared with control flies, lower levels of TAG were also found in the *dilp7* mutants. The effects of *dilp7* knockout on lipid metabolism were also indirectly confirmed by recent data showing that down-regulation of insulin signaling led to reduced lipid accumulation (Linneweber et al., 2014). In previous studies, ablation of insulin-producing cells either resulted in no change in lipid content (Broughton et al., 2008) or slightly increased it (Broughton et al., 2005).

## General picture and conclusions

The present study explored the influence of *dilp* knockouts on (1) food intake and (2) levels of stored and circulating metabolites. The functions of DILPs in *Drosophila* resemble those of insulin in mammals, namely the lowering of glucose levels in blood (hemolymph in insects) by increasing glucose uptake by cells and directing it into production of storage metabolites, including glycogen and lipids. Our current data showed that the balance of dietary macronutrients (proteins and carbohydrates) influences the outcomes of an insulin-like peptide deficiency. Moreover, we have shown that knockouts of specific insulin-like peptides of *Drosophila* affect feeding behavior. It was clearly shown that different DILPs mediate regulation of particular aspects of diet-dependent metabolism. From the current data, we show that DILP2 and DILP5 can be involved in diet-independent accumulation of glycogen. DILP3 was shown to influence trehalose synthesis and/or release into the hemolymph, whereas DILP5 and DILP7 mainly affected TAG synthesis on high carbohydrate diets. These specific roles for DILPs are likely conferred by nutrient-dependent DILP release by neurosecretory cells and specific target cells for DILPs (Kim and Neufeld, 2015). The specific connection between feeding behavior and DILPs, found in the current study, may reflect an impact of nutrient cues on signaling effects of DILPs. In other words, specific ratios of macronutrients can evoke DILP release and subsequent regulation of metabolism in particular tissues.

## Author contributions

OL, KS, and SS: conceptualization; OL and SS: methodology; US, KF, and IY: investigation; DG, OL, and UV: writing—original draft, OL, SS, KS, and AV: writing—reviews and editing; US, OL, and DG: formal analysis, SS: data curation; OL, DG, and SS: visualization.

### Conflict of interest statement

The authors declare that the research was conducted in the absence of any commercial or financial relationships that could be construed as a potential conflict of interest.

## Abbreviations

DILPs: *Drosophila* insulin-like peptides
dInR: *Drosophila* insulin receptor
HG: Hemolymph glucose
HT: trehalose in hemolymph
TAG: triacylglyceride
Y: yeast autolysate solution.
