# Supplementary material for: Insulin-Like Peptides Regulate Feeding Preference and Metabolism in Drosophila
Source: Front Physiol. 2018 Aug 24;9:1083. doi: 10.3389/fphys.2018.01083 (PMC6118219; doi:10.3389/fphys.2018.01083)
Supplement: Supplementary file 1 [file Table_1.pdf]

SUPPLEMENTAL DATA

Semaniuk UV, Gospodaryov DV, Feden'ko KM, Yurkevych IS, Vaiserman AM, Storey KB, Simpson SJ and Lushchak O (2018) Insulin-like peptides regulate feeding preference and metabolism in *Drosophila*. Front. Physiol. 9:1083. doi: 10.3389/fphys.2018.01083

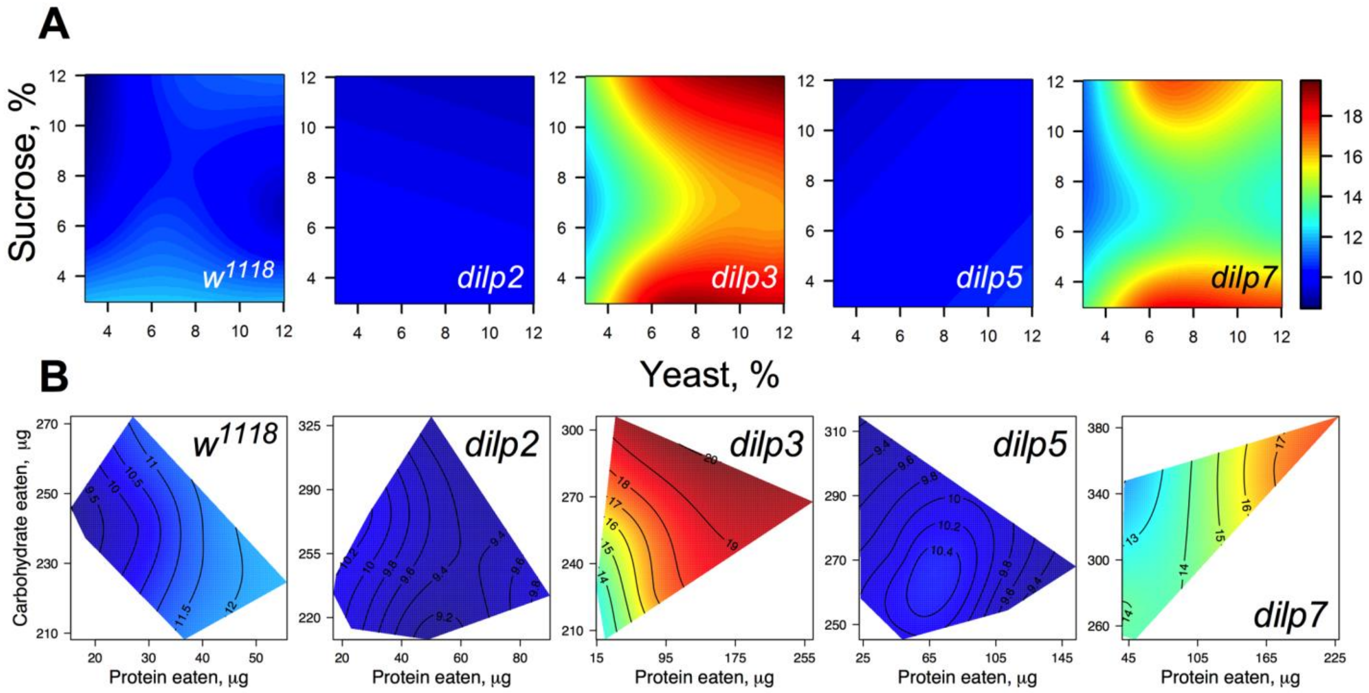

Figure S1. Diet-dependent patterns of body glucose in the wild type and *dilp* mutant flies. (A) Dietary response surfaces depicting dependence of glucose content in the body of individuals of the the wild type (w^{1118}) and *dilp* mutant lines on concentrations of yeast and sucrose in the diet. (B) Dietary response surfaces showing dependence of body glucose content in the the wild type and *dilp* mutant lines on amounts of protein and carbohydrate consumed. Each surface has own scale shown by contour lines. Data are n = 3-6 replicates for each diet and fly line combination.

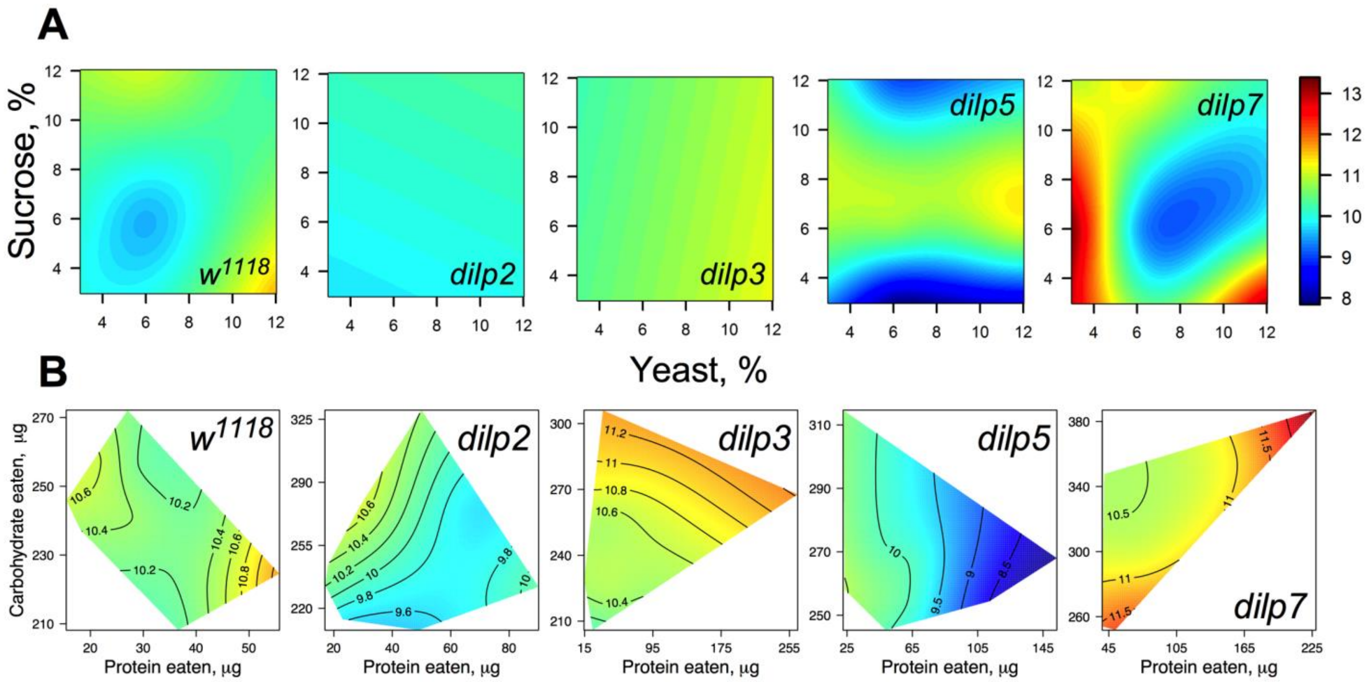

Figure S2. Diet-dependent patterns of body trehalose in the wild type and *dilp* mutant flies. (A) Dietary response surfaces depicting dependence of trehalose content in the body of individuals of the wild type (w^{1118}) and *dilp* mutant lines on concentrations of yeast and sucrose in the diet. (B) Dietary response surfaces showing dependence of body trehalose content in the wild type and *dilp* mutant lines on amounts of protein and carbohydrate consumed. Each surface has own scale shown by contour lines. Data are n = 3-6 replicates for each diet and fly line combination.

Table S1. Genotypes and stock numbers of fruit fly mutant lines used in this study.

Name	Genotype	Genetic background	Bloomington stock number	Cytogenetic location of the mutation
dilp1 mutant	*w**; $dilp1^{1}$	w^{1118}	78055	3L (67C8)
dilp2 mutant	*w**; $dilp2^{1}$	w^{1118}	30881	3L (67C8)
dilp3 mutant	*w**; $dilp3^{1}$	w^{1118}	30882	3L (67C8)
dilp4 mutant	*w**; $dilp4^{1}$	w^{1118}	30883	3L (67C8)
dilp5 mutant	*w**; $dilp5^{1}$	w^{1118}	30885	3L (67C9)
dilp7 mutant	*w**, $dilp7^{1}$	w^{1118}	30887	X (3E2)
dilp2-3 mutant	*w**; $dilp2\text{-}3^{1}$	w^{1118}	30889	3L (67C8)

Datasheet S2. Summary for two-way analysis of variance performed on data of food consumption by the wild type and *dilp* mutants (Figure 1 and Figure 2). Asterisk denotes statistical significance of the estimate.

Datasheet S3. Significance of differences (*p*-values) between consumption of food by the wild type and *dilp* mutants on different diets (Figure 1 and Figure 2). The *post hoc* analysis was performed by Tukey's honest significant difference test with Bonferroni correction. Gray color shows significance of the difference.

Datasheet S4. Significance of differences (marked $p < 0.05$) between values for the wild type and mutant lines on particular diets. The *post hoc* analysis was performed by Tukey's honest significant difference test with Bonferroni correction. "1" highlighted by gray color means significant difference between value for individuals of the wild type line w^{1118} and the mutant for corresponding diet. The data were analysed by one-way ANOVA followed by Tukey's honest significant difference test with Bonferroni correction.

Table S5. Coefficients for genotype-diet interactions provided by generalized linear models assessing contribution of genotype to volume of sucrose solution ingested or amount of sucrose eaten. Asterisk denotes statistical significance of the interaction.

	Sucrose volume					Sucrose amount				
	value	df	χ^2	*p*-value		value	df	χ^2	*p*-value	
w^{1118}-dilp1	0.286	1.00	1.09	1.000		21.622	1.00	7.29	0.083	
w^{1118}-dilp2	0.005	1.00	0.00	1.000		-12.640	1.00	2.76	0.775	
w^{1118}-dilp2,3	-0.239	1.00	0.88	1.000		-34.652	1.00	21.64	0.000	*
w^{1118}-dilp3	-0.378	1.00	2.11	1.000		-4.101	1.00	0.29	1.000	
w^{1118}-dilp4	0.194	1.00	0.41	1.000		-27.167	1.00	9.39	0.033	*
w^{1118}-dilp5	0.090	1.00	0.12	1.000		-25.253	1.00	11.44	0.012	*
w^{1118}-dilp7	0.752	1.00	7.54	0.157		-60.351	1.00	56.77	0.000	*
dilp1-dilp2	-0.281	1.00	0.84	1.000		-34.262	1.00	14.58	0.003	*
dilp1-dilp2,3	-0.525	1.00	3.02	1.000		-56.274	1.00	40.58	0.000	*
dilp1-dilp3	-0.092	1.00	0.09	1.000		-25.723	1.00	8.22	0.054	
dilp1-dilp4	0.480	1.00	1.95	1.000		-48.789	1.00	23.53	0.000	*
dilp1-dilp5	0.376	1.00	1.54	1.000		-46.875	1.00	28.07	0.000	*
dilp1-dilp7	1.037	1.00	10.62	0.030	*	-81.973	1.00	77.50	0.000	*
dilp2-dilp2,3	-0.244	1.00	0.71	1.000		-22.013	1.00	6.75	0.103	
dilp2-dilp3	-0.373	1.00	1.60	1.000		8.538	1.00	0.98	1.000	
dilp2-dilp4	0.199	1.00	0.36	1.000		-14.527	1.00	2.22	0.952	
dilp2-dilp5	0.095	1.00	0.11	1.000		-12.613	1.00	2.21	0.952	
dilp2-dilp7	0.757	1.00	6.08	0.342		-47.711	1.00	28.28	0.000	*
dilp2,3-dilp3	-0.617	1.00	4.53	0.764		30.551	1.00	12.99	0.006	*
dilp2,3-dilp4	-0.045	1.00	0.02	1.000		7.486	1.00	0.61	1.000	
dilp2,3-dilp5	-0.149	1.00	0.27	1.000		9.400	1.00	1.27	1.000	
dilp2,3-dilp7	0.513	1.00	2.88	1.000		-25.699	1.00	8.46	0.051	
dilp3-dilp4	-0.572	1.00	2.95	1.000		-23.066	1.00	5.60	0.161	
dilp3-dilp5	-0.468	1.00	2.60	1.000		-21.151	1.00	6.21	0.127	
dilp3-dilp7	-1.130	1.00	13.56	0.006	*	-56.250	1.00	39.30	0.000	*
dilp4-dilp5	0.104	1.00	0.10	1.000		1.914	1.00	0.04	1.000	
dilp4-dilp7	-0.558	1.00	2.63	1.000		-33.184	1.00	10.89	0.015	*
dilp5-dilp7	-0.662	1.00	4.78	0.689		-35.098	1.00	15.74	0.001	*

Table S6. Coefficients for genotype-diet interactions provided by generalized linear models assessing contribution of genotype to volume of yeast autolysate solution ingested or amount of yeast autolysate eaten. Asterisk denotes statistical significance of the interaction.

	Yeast autolysate volume					Yeast autolysate amount				
	value	df	χ^2	*p*-value		value	df	χ^2	*p*-value	
w^{1118}-dilp1	-0.091	1.00	0.31	1.000		-1.219	1.00	0.04	1.000	
w^{1118}-dilp2	-0.121	1.00	0.60	1.000		-10.526	1.00	3.60	0.404	
w^{1118}-dilp2,3	-1.590	1.00	107.89	0.000	*	-50.307	1.00	85.96	0.000	*
w^{1118}-dilp3	-0.776	1.00	24.57	0.000	*	-32.376	1.00	34.08	0.000	*
w^{1118}-dilp4	-0.914	1.00	25.18	0.000	*	-25.669	1.00	15.80	0.001	*
w^{1118}-dilp5	-1.137	1.00	54.89	0.000	*	-35.446	1.00	42.48	0.000	*
w^{1118}-dilp7	-1.959	1.00	141.61	0.000	*	-55.870	1.00	91.70	0.000	*
dilp1-dilp2	-0.030	1.00	0.03	1.000		-9.307	1.00	2.03	0.927	
dilp1-dilp2,3	-1.499	1.00	68.18	0.000	*	-49.088	1.00	58.20	0.000	*
dilp1-dilp3	-0.684	1.00	13.78	0.002	*	-31.158	1.00	22.73	0.000	*
dilp1-dilp4	-0.823	1.00	15.87	0.001		-24.450	1.00	11.14	0.010	*
dilp1-dilp5	-1.046	1.00	33.07	0.000	*	-34.227	1.00	28.20	0.000	*
dilp1-dilp7	-1.868	1.00	95.27	0.000	*	-54.651	1.00	64.93	0.000	*
dilp2-dilp2,3	-1.469	1.00	71.16	0.000	*	-39.781	1.00	41.52	0.000	*
dilp2-dilp3	-0.655	1.00	13.66	0.002	*	-21.850	1.00	12.11	0.007	*
dilp2-dilp4	-0.794	1.00	15.71	0.001	*	-15.143	1.00	4.55	0.263	
dilp2-dilp5	-1.016	1.00	33.90	0.000	*	-24.919	1.00	16.23	0.001	*
dilp2-dilp7	-1.838	1.00	99.37	0.000	*	-45.344	1.00	48.13	0.000	*
dilp2,3-dilp3	0.814	1.00	21.86	0.000	*	17.930	1.00	8.43	0.037	*
dilp2,3-dilp4	0.676	1.00	11.69	0.006	*	24.638	1.00	12.37	0.006	*
dilp2,3-dilp5	0.453	1.00	6.99	0.066		14.861	1.00	5.98	0.130	
dilp2,3-dilp7	-0.369	1.00	4.13	0.269		-5.563	1.00	0.75	1.000	
dilp3-dilp4	-0.139	1.00	0.48	1.000		6.707	1.00	0.89	1.000	
dilp3-dilp5	-0.361	1.00	4.28	0.269		-3.069	1.00	0.25	1.000	
dilp3-dilp7	-1.183	1.00	41.18	0.000	*	-23.493	1.00	12.92	0.005	*
dilp4-dilp5	-0.222	1.00	1.26	1.000		-9.777	1.00	1.94	0.927	
dilp4-dilp7	-1.044	1.00	25.54	0.000	*	-30.201	1.00	16.99	0.001	*
dilp5-dilp7	-0.822	1.00	20.44	0.000	*	-20.424	1.00	10.04	0.017	*

Table S7. Coefficients of the generalized additive models describing the response of circulating glucose in fruit fly lines investigated in this study to concentrations of sucrose and yeast in food. Here and in the tables below: edf – estimated degrees of freedom; ref. df – degrees of freedom for reference distribution. *Significance of smooth terms fit by F-test ($p < 0.05$).

	w^{1118}	*dilp2*	*dilp3*	*dilp5*	*dilp7*
edf					
S	0.7843	1.3690	0.0001	0.7418	0.0001
Y	0.0001	0.5779	1.5576	0.6503	1.3210
S × Y	0.7292	0.0000	0.4737	0.0001	1.4120
ref. df					
S	2.0	2.0	2.0	2.0	2.0
Y	2.0	2.0	2.0	2.0	2.0
S × Y	3.0	3.0	3.0	3.0	3.0
F					
S	1.817	1.969	0.000	1.436	0.000
Y	0.000	0.685	6.006	0.930	2.830
S × Y	0.507	0.000	0.214	0.000	1.331
p-value					
S	0.0261	0.0711	0.4778	0.0572	0.7685
Y	0.6007*	0.1324	0.0011*	0.1000	0.0117*
S × Y	0.1299	0.5411	0.2474	0.5014	0.0528

Table S8. Coefficients of the generalized additive models describing the response of circulating trehalose in fruit fly lines investigated in this study to concentrations of sucrose and yeast in food. Other information as in Table S7.

	w^{1118}	*dilp2*	*dilp3*	*dilp5*	*dilp7*
edf					
S	0.0001	0.8971	0.9713	1.4534	0.1176
Y	0.9170	0.0000	0.9288	0.5652	0.9494
S × Y	1.3658	1.3630	0.0000	0.1751	1.3707
ref. df					
S	2.0	2.0	2.0	2.0	2.0
Y	2.0	2.0	2.0	2.0	2.0
S × Y	3.0	3.0	3.0	3.0	3.0
F					
S	0.000	4.359	6.524	2.398	0.067
Y	5.523	0.000	16.894	0.650	9.383
S × Y	2.162	2.829	0.000	0.068	1.761
p-value					
S	0.5574	0.0017*	0.0000*	0.0486*	0.2903
Y	0.0010*	0.9517	0.0007*	0.1373	0.0001*
S × Y	0.0134*	0.0054*	0.8788	0.2803	0.0316*

Table S9. Coefficients of the generalized additive models describing the response of glycogen content in fruit fly lines investigated in this study to concentrations of sucrose and yeast in food. Other information as in Table S7.

	w[1118]	*dilp2*	*dilp3*	*dilp5*	*dilp7*
edf					
S	1.5600	0.0000	1.6260	0.0001	0.9041
Y	0.0000	0.0000	0.5468	1.3000	0.0001
S × Y	0.0000	0.0000	0.0000	1.2170	1.6031
Ref. df					
S	2.0	2.0	2.0	2.0	2.0
Y	2.0	2.0	2.0	2.0	2.0
S × Y	3.0	3.0	3.0	3.0	3.0
F					
S	5.629	0.000	12.159	0.000	4.714
Y	0.000	0.000	0.603	2.593	0.000
S × Y	0.000	0.000	0.000	1.538	3.423
p-value					
S	0.0018*	1.0000	0.0000*	0.5807	0.0000*
Y	0.9731	1.0000	0.1470	0.0270*	0.4413
S × Y	0.9995	1.0000	0.4620	0.0308*	0.0036*

Table S10. Coefficients of the generalized additive models describing the response of triacylglyceride content in fruit fly lines investigated in this study to concentrations of sucrose and yeast in food. Other information as in Table S7.

	w[1118]	*dilp2*	*dilp3*	*dilp5*	*dilp7*
edf					
S	0.9335	0.9445	0.0000	0.0000	0.8101
Y	0.0000	0.8561	0.4947	0.5962	0.8291
S × Y	0.2901	0.0001	0.0000	0.0003	2.1608
Ref. df					
S	2.0	2.0	2.0	2.0	2.0
Y	2.0	2.0	2.0	2.0	2.0
S × Y	3.0	3.0	3.0	3.0	3.0
F					
S	7.016	8.507	0.000	0.000	2.133
Y	0.000	2.973	0.489	0.738	2.424
S × Y	0.122	0.000	0.000	0.000	2.811
p-value					
S	0.0003*	0.0001*	0.8060	1.0000	0.0260*
Y	0.9351	0.0123*	0.1680	0.1250	0.0194*
S × Y	0.2753	0.5186	0.8980	0.3560	0.0168*

Table S11. Coefficients of the generalized additive models describing the response of whole body glucose content in fruit fly lines investigated in this study to concentrations of sucrose and yeast in food. Other information as in Table S7.

	w^{1118}	*dilp2*	*dilp3*	*dilp5*	*dilp7*
edf					
S	1.5269	0.1836	0.7189	0.3512	1.6990
Y	0.0003	0.0000	1.4840	0.0001	1.7200
S × Y	0.3992	0.0000	0.0000	0.0000	0.0001
Ref. df					
S	2.0	2.0	2.0	2.0	2.0
Y	2.0	2.0	2.0	2.0	2.0
S × Y	3.0	3.0	3.0	3.0	3.0
F					
S	2.521	0.112	1.278	0.271	5.060
Y	0.000	0.000	3.451	0.000	4.566
S × Y	0.189	0.000	0.000	0.000	0.000
p-value					
S	0.0411*	0.2760	0.0680	0.2230	0.0064*
Y	0.4442	0.6950	0.0179*	0.3970	0.0102*
S × Y	0.2262	0.8050	0.5661	0.8610	0.6891

Table S12. Coefficients of the generalized additive models describing the response of whole body trehalose content in fruit fly lines investigated in this study to concentrations of sucrose and yeast in food. Other information as in Table S7.

	w^{1118}	*dilp2*	*dilp3*	*dilp5*	*dilp7*
edf					
S	0.0000	0.0000	0.0000	0.5899	0.0011
Y	0.0000	0.0000	0.0001	0.0006	0.0002
S × Y	0.7184	0.0001	0.0000	0.0000	0.0001
Ref. df					
S	2.0	2.0	2.0	2.0	2.0
Y	2.0	2.0	2.0	2.0	2.0
S × Y	3.0	3.0	3.0	3.0	3.0
F					
S	0.000	0.000	0.000	0.719	0.001
Y	0.000	0.000	0.000	0.000	0.000
S × Y	0.458	0.000	0.000	0.000	0.000
p-value					
S	0.7580	0.8090	0.9200	0.1280	0.3300
Y	0.6210	0.9720	0.3940	0.3340	0.4080
S × Y	0.1580	0.4300	0.4300	0.6320	0.7920

Table S13. Coefficients of the generalized additive models describing the response of weight of fruit fly lines investigated in this study to concentrations of sucrose and yeast in food. Other information as in Table S7.

	w^{1118}	*dilp2*	*dilp3*	*dilp5*	*dilp7*
edf					
S	0.8210	0.8143	0.0000	0.0000	0.0000
Y	0.7545	0.0000	0.5382	0.0000	0.0000
S × Y	0.0000	0.0000	0.0000	0.0000	0.6730
Ref. df					
S	2.0	2.0	2.0	2.0	2.0
Y	2.0	2.0	2.0	2.0	2.0
S × Y	3.0	3.0	3.0	3.0	3.0
F					
S	2.294	1.414	0.583	0.000	0.000
Y	1.536	0.000	0.000	0.000	0.000
S × Y	0.000	0.000	0.000	0.000	0.381
p-value					
S	0.0218*	0.0610	0.1500	0.7940	0.4670
Y	0.0487*	0.9750	0.9490	0.5910	0.8710
S × Y	0.9404	0.6710	0.6790	1.0000	0.1940
