# Supplementary material for: Insulin-Like Peptides Regulate Feeding Preference and Metabolism in Drosophila
Source: Front Physiol. 2018 Aug 24;9:1083. doi: 10.3389/fphys.2018.01083 (PMC6118219; doi:10.3389/fphys.2018.01083)
Supplement: Supplementary file 4 [file Data_Sheet_4.DOC]

Significance of differences between values for wild type wild type and mutant lines. “1” highlighted by gray color means significant difference between value for individuals of wild type line *w^1118^* and the mutant for corresponding diet. The data were analysed by one-way ANOVA followed by Tukey’s honest significant difference test with Bonferroni correction.

Hemolymph Glucose

|  | *dilp2* |  | | | *dilp3* |  | | | *dilp5* |  | | | | *dilp7* | |  | | |
| --- | --- | --- | --- | --- | --- | --- | --- | --- | --- | --- | --- | --- | --- | --- | --- | --- | --- | --- |
| Sucrose | 12 |  |  |  | 12 |  | 1 |  | 12 | | 1 |  |  | 12 |  | |  |  |
|  | 6 |  | 1 |  | 6 |  |  |  | 6 | |  |  |  | 6 |  | |  |  |
|  | 3 |  |  |  | 3 |  | 1 |  | 3 | |  |  | 1 | 3 |  | | 1 |  |
|  |  | 3 | 6 | 12 |  | 3 | 6 | 12 |  | | 3 | 6 | 12 |  | 3 | | 6 | 12 |
|  |  | Yeast | | |  | Yeast | | |  | | Yeast | | |  | Yeast | | | |

Hemolymph Trehalose

|  | *dilp2* |  | | | *dilp3* |  | | | *dilp5* |  | | | | *dilp7* | |  | | |
| --- | --- | --- | --- | --- | --- | --- | --- | --- | --- | --- | --- | --- | --- | --- | --- | --- | --- | --- |
| Sucrose | 12 |  |  |  | 12 | 1 | 1 | 1 | 12 | |  |  |  | 12 |  | |  |  |
|  | 6 | 1 |  |  | 6 | 1 | 1 | 1 | 6 | |  |  |  | 6 |  | |  |  |
|  | 3 |  |  |  | 3 |  | 1 | 1 | 3 | |  |  |  | 3 |  | |  |  |
|  |  | 3 | 6 | 12 |  | 3 | 6 | 12 |  | | 3 | 6 | 12 |  | 3 | | 6 | 12 |
|  |  | Yeast | | |  | Yeast | | |  | | Yeast | | |  | Yeast | | | |

Body Glucose

|  | *dilp2* |  | | | *dilp3* |  | | | *dilp5* |  | | | | *dilp7* | |  | | |
| --- | --- | --- | --- | --- | --- | --- | --- | --- | --- | --- | --- | --- | --- | --- | --- | --- | --- | --- |
| Sucrose | 12 |  |  |  | 12 | 1 | 1 | 1 | 12 | |  |  |  | 12 | 1 | | 1 | 1 |
|  | 6 |  |  |  | 6 |  |  | 1 | 6 | |  |  |  | 6 |  | |  |  |
|  | 3 |  | 1 |  | 3 |  | 1 | 1 | 3 | |  | 1 |  | 3 |  | | 1 | 1 |
|  |  | 3 | 6 | 12 |  | 3 | 6 | 12 |  | | 3 | 6 | 12 |  | 3 | | 6 | 12 |
|  |  | Yeast | | |  | Yeast | | |  | | Yeast | | |  | Yeast | | | |

Body Trehalose

|  | *dilp2* |  | | | *dilp3* |  | | | *dilp5* |  | | | | *dilp7* | |  | | |
| --- | --- | --- | --- | --- | --- | --- | --- | --- | --- | --- | --- | --- | --- | --- | --- | --- | --- | --- |
| Sucrose | 12 |  |  |  | 12 |  |  |  | 12 | |  |  |  | 12 |  | |  |  |
|  | 6 |  |  |  | 6 |  |  |  | 6 | |  |  |  | 6 |  | |  |  |
|  | 3 |  |  |  | 3 |  |  |  | 3 | |  |  |  | 3 |  | |  |  |
|  |  | 3 | 6 | 12 |  | 3 | 6 | 12 |  | | 3 | 6 | 12 |  | 3 | | 6 | 12 |
|  |  | Yeast | | |  | Yeast | | |  | | Yeast | | |  | Yeast | | | |

Glycogen

|  | *dilp2* |  | | | *dilp3* |  | | | *dilp5* |  | | | | *dilp7* | |  | | |
| --- | --- | --- | --- | --- | --- | --- | --- | --- | --- | --- | --- | --- | --- | --- | --- | --- | --- | --- |
| Sucrose | 12 | 1 | 1 | 1 | 12 | 1 |  | 1 | 12 | | 1 | 1 | 1 | 12 | 1 | |  |  |
|  | 6 | 1 | 1 | 1 | 6 | 1 |  |  | 6 | |  |  | 1 | 6 |  | | 1 |  |
|  | 3 | 1 | 1 | 1 | 3 |  | 1 |  | 3 | |  | 1 | 1 | 3 |  | | 1 | 1 |
|  |  | 3 | 6 | 12 |  | 3 | 6 | 12 |  | | 3 | 6 | 12 |  | 3 | | 6 | 12 |
|  |  | Yeast | | |  | Yeast | | |  | | Yeast | | |  | Yeast | | | |

TAG

|  | *dilp2* |  | | | *dilp3* |  | | | *dilp5* |  | | | | *dilp7* | |  | | |
| --- | --- | --- | --- | --- | --- | --- | --- | --- | --- | --- | --- | --- | --- | --- | --- | --- | --- | --- |
| Sucrose | 12 |  |  |  | 12 |  | 1 |  | 12 | | 1 | 1 | 1 | 12 | 1 | | 1 |  |
|  | 6 |  |  |  | 6 |  |  |  | 6 | |  | 1 | 1 | 6 | 1 | |  | 1 |
|  | 3 |  |  |  | 3 |  |  |  | 3 | |  | 1 |  | 3 | 1 | |  | 1 |
|  |  | 3 | 6 | 12 |  | 3 | 6 | 12 |  | | 3 | 6 | 12 |  | 3 | | 6 | 12 |
|  |  | Yeast | | |  | Yeast | | |  | | Yeast | | |  | Yeast | | | |

Weight

|  | *dilp2* |  | | | *dilp3* |  | | | *dilp5* |  | | | | *dilp7* | |  | | |
| --- | --- | --- | --- | --- | --- | --- | --- | --- | --- | --- | --- | --- | --- | --- | --- | --- | --- | --- |
| Sucrose | 12 |  | 1 |  | 12 |  |  |  | 12 | |  | 1 |  | 12 |  | |  |  |
|  | 6 |  |  |  | 6 |  |  |  | 6 | | 1 |  |  | 6 |  | |  |  |
|  | 3 |  |  |  | 3 |  |  |  | 3 | |  |  |  | 3 |  | |  |  |
|  |  | 3 | 6 | 12 |  | 3 | 6 | 12 |  | | 3 | 6 | 12 |  | 3 | | 6 | 12 |
|  |  | Yeast | | |  | Yeast | | |  | | Yeast | | |  | Yeast | | | |

Sucrose volume

|  | *dilp2* |  | | | | | *dilp3* |  | | | | | *dilp5* |  | | | | | | *dilp7* | |  | | |
| --- | --- | --- | --- | --- | --- | --- | --- | --- | --- | --- | --- | --- | --- | --- | --- | --- | --- | --- | --- | --- | --- | --- | --- | --- |
| Sucrose | 12 |  |  | | 1 | | 12 |  |  | |  | | 12 | | 1 |  | |  | | 12 | 1 | |  |  |
|  | 6 |  |  | |  | | 6 |  |  | |  | | 6 | |  |  | |  | | 6 |  | |  |  |
|  | 3 |  |  | |  | | 3 |  |  | | 1 | | 3 | |  |  | |  | | 3 |  | | 1 | 1 |
|  |  | 3 | 6 | | 12 | |  | 3 | 6 | | 12 | |  | | 3 | 6 | | 12 | |  | 3 | | 6 | 12 |
|  |  | Yeast | | | | |  | Yeast | | | | |  | | Yeast | | | | |  | Yeast | | | |
|  |  |  | | | | |  |  | | | | |  | |  | | | | |  |  | | | |
|  | *dilp1* |  | | | | | *dilp2,3* |  | | | | | *dilp4* | |  | | | | |  |  | | | |
| Sucrose | 12 |  | | 1 | | 1 | 12 |  | |  | |  | 12 | | 1 | |  | |  |  |  | | | |
|  | 6 |  | |  | |  | 6 |  | |  | |  | 6 | |  | |  | |  |  |  | | | |
|  | 3 | 1 | |  | |  | 3 |  | |  | |  | 3 | |  | |  | |  |  |  | | | |
|  |  | 3 | | 6 | | 12 |  | 3 | | 6 | | 12 |  | | 3 | | 6 | | 12 |  |  | | | |
|  |  | Yeast | | | | |  | Yeast | | | | |  | | Yeast | | | | |  |  | | | |

Yeast volume

|  | *dilp2* |  | | | | | *dilp3* |  | | | | | *dilp5* |  | | | | | | *dilp7* | |  | | |
| --- | --- | --- | --- | --- | --- | --- | --- | --- | --- | --- | --- | --- | --- | --- | --- | --- | --- | --- | --- | --- | --- | --- | --- | --- |
| Sucrose | 12 |  |  | | 1 | | 12 | 1 |  | | 1 | | 12 | | 1 | 1 | | 1 | | 12 | 1 | | 1 | 1 |
|  | 6 |  | 1 | | 1 | | 6 | 1 |  | | 1 | | 6 | |  | 1 | | 1 | | 6 | 1 | | 1 | 1 |
|  | 3 | 1 | 1 | | 1 | | 3 |  | 1 | | 1 | | 3 | | 1 | 1 | | 1 | | 3 | 1 | | 1 | 1 |
|  |  | 3 | 6 | | 12 | |  | 3 | 6 | | 12 | |  | | 3 | 6 | | 12 | |  | 3 | | 6 | 12 |
|  |  | Yeast | | | | |  | Yeast | | | | |  | | Yeast | | | | |  | Yeast | | | |
|  |  |  | | | | |  |  | | | | |  | |  | | | | |  |  | | | |
|  | *dilp1* |  | | | | | *dilp2,3* |  | | | | | *dilp4* | |  | | | | |  |  | | | |
| Sucrose | 12 | 1 | |  | |  | 12 | 1 | | 1 | | 1 | 12 | | 1 | | 1 | | 1 |  |  | | | |
|  | 6 | 1 | |  | |  | 6 | 1 | | 1 | | 1 | 6 | | 1 | |  | | 1 |  |  | | | |
|  | 3 | 1 | |  | |  | 3 | 1 | | 1 | | 1 | 3 | | 1 | |  | | 1 |  |  | | | |
|  |  | 3 | | 6 | | 12 |  | 3 | | 6 | | 12 |  | | 3 | | 6 | | 12 |  |  | | | |
|  |  | Yeast | | | | |  | Yeast | | | | |  | | Yeast | | | | |  |  | | | |

Sucrose amount

|  | *dilp2* |  | | | | | *dilp3* |  | | | | | *dilp5* |  | | | | | | *dilp7* | |  | | |
| --- | --- | --- | --- | --- | --- | --- | --- | --- | --- | --- | --- | --- | --- | --- | --- | --- | --- | --- | --- | --- | --- | --- | --- | --- |
| Sucrose | 12 |  |  | | 1 | | 12 |  |  | |  | | 12 | | 1 |  | |  | | 12 |  | | 1 | 1 |
|  | 6 |  |  | |  | | 6 |  |  | |  | | 6 | |  |  | |  | | 6 |  | |  | 1 |
|  | 3 |  |  | |  | | 3 |  |  | | 1 | | 3 | | 1 | 1 | |  | | 3 | 1 | |  |  |
|  |  | 3 | 6 | | 12 | |  | 3 | 6 | | 12 | |  | | 3 | 6 | | 12 | |  | 3 | | 6 | 12 |
|  |  | Yeast | | | | |  | Yeast | | | | |  | | Yeast | | | | |  | Yeast | | | |
|  |  |  | | | | |  |  | | | | |  | |  | | | | |  |  | | | |
|  | *dilp1* |  | | | | | *dilp2,3* |  | | | | | *dilp4* | |  | | | | |  |  | | | |
| Sucrose | 12 |  | |  | | 1 | 12 |  | |  | |  | 12 | | 1 | |  | |  |  |  | | | |
|  | 6 |  | |  | |  | 6 |  | |  | |  | 6 | |  | |  | | 1 |  |  | | | |
|  | 3 | 1 | |  | |  | 3 |  | | 1 | | 1 | 3 | |  | |  | | 1 |  |  | | | |
|  |  | 3 | | 6 | | 12 |  | 3 | | 6 | | 12 |  | | 3 | | 6 | | 12 |  |  | | | |
|  |  | Yeast | | | | |  | Yeast | | | | |  | | Yeast | | | | |  |  | | | |

Yeast amount

|  | *dilp2* |  | | | | | *dilp3* |  | | | | | *dilp5* |  | | | | | | *dilp7* | |  | | |
| --- | --- | --- | --- | --- | --- | --- | --- | --- | --- | --- | --- | --- | --- | --- | --- | --- | --- | --- | --- | --- | --- | --- | --- | --- |
| Sucrose | 12 |  |  | | 1 | | 12 | 1 |  | | 1 | | 12 | | 1 | 1 | | 1 | | 12 | 1 | | 1 | 1 |
|  | 6 |  | 1 | | 1 | | 6 | 1 |  | | 1 | | 6 | |  | 1 | | 1 | | 6 | 1 | | 1 | 1 |
|  | 3 | 1 | 1 | | 1 | | 3 |  | 1 | | 1 | | 3 | | 1 | 1 | | 1 | | 3 | 1 | | 1 | 1 |
|  |  | 3 | 6 | | 12 | |  | 3 | 6 | | 12 | |  | | 3 | 6 | | 12 | |  | 3 | | 6 | 12 |
|  |  | Yeast | | | | |  | Yeast | | | | |  | | Yeast | | | | |  | Yeast | | | |
|  |  |  | | | | |  |  | | | | |  | |  | | | | |  |  | | | |
|  | *dilp1* |  | | | | | *dilp2,3* |  | | | | | *dilp4* | |  | | | | |  |  | | | |
| Sucrose | 12 | 1 | |  | |  | 12 | 1 | | 1 | | 1 | 12 | | 1 | | 1 | | 1 |  |  | | | |
|  | 6 | 1 | |  | |  | 6 | 1 | | 1 | | 1 | 6 | | 1 | |  | | 1 |  |  | | | |
|  | 3 | 1 | |  | |  | 3 | 1 | | 1 | | 1 | 3 | | 1 | |  | | 1 |  |  | | | |
|  |  | 3 | | 6 | | 12 |  | 3 | | 6 | | 12 |  | | 3 | | 6 | | 12 |  |  | | | |
|  |  | Yeast | | | | |  | Yeast | | | | |  | | Yeast | | | | |  |  | | | |
